# Resolving fluorescent species by their brightness and diffusion using correlated photon-counting histograms

**DOI:** 10.1371/journal.pone.0226063

**Published:** 2019-12-30

**Authors:** Nathan Scales, Peter S. Swain

**Affiliations:** 1 Department of Physiology, McGill University, 3655 Promenade Sir William Osler, Montreal, Quebec H3G 1Y6, Canada; 2 School of Biological Sciences, University of Edinburgh, Mayfield Road, Edinburgh EH9 3BF, United Kingdom; Indian Institute of Technology Kanpur, INDIA

## Abstract

Fluorescence fluctuation spectroscopy (FFS) refers to techniques that analyze fluctuations in the fluorescence emitted by fluorophores diffusing in a small volume and can be used to distinguish between populations of molecules that exhibit differences in brightness or diffusion. For example, fluorescence correlation spectroscopy (FCS) resolves species through their diffusion by analyzing correlations in the fluorescence over time; photon counting histograms (PCH) and related methods based on moment analysis resolve species through their brightness by analyzing fluctuations in the photon counts. Here we introduce correlated photon counting histograms (cPCH), which uses both types of information to simultaneously resolve fluorescent species by their brightness and diffusion. We define the cPCH distribution by the probability to detect both a particular number of photons at the current time and another number at a later time. FCS and moment analysis are special cases of the moments of the cPCH distribution, and PCH is obtained by summing over the photon counts in either channel. cPCH is inherently a dual channel technique, and the expressions we develop apply to the dual colour case. Using simulations, we demonstrate that two species differing in both their diffusion and brightness can be better resolved with cPCH than with either FCS or PCH. Further, we show that cPCH can be extended both to longer dwell times to improve the signal-to-noise and to the analysis of images. By better exploiting the information available in fluorescence fluctuation spectroscopy, cPCH will be an enabling methodology for quantitative biology.

## Introduction

Absolute numbers are necessary for quantitative biology, but are challenging to obtain in single cells. Although *ad hoc* techniques exist [1–6], an established approach to measure the concentrations of fluorescently tagged molecules and their rate of diffusion is fluorescence fluctuation spectroscopy, which has two main branches. Fluorescence-correlation spectroscopy (FCS) uses correlations between the observed photon counts at different times to obtain both the diffusion coefficient and the local numbers of a fluorescent molecule in the illuminated observation volume [7]. Analysis of photon-counting histograms [8, 9] and related methods of moments [10, 11] exploit fluctuations in the amplitude of the photon counts to estimate the brightness of a fluorescent molecule (and so allow an estimate of absolute numbers and the degree of oligomerization through the number of fluorescence tags per molecule). Nevertheless, information on both diffusion and brightness are often needed [12, 13]. Although it is possible to employ PCH and FCS on the same data [12–14], the two techniques are not inherently linked, either making interpretation complicated or requiring global fits across multiple experiments to extract the parameters of interest.

Techniques developed to be sensitive to both diffusion and brightness do exist, but often are either computationally intensive or employ approximations that can limit their use. For example, higher-order FCS [15] does not consider the statistical uncertainty of the higher order moments and even later implementations are not able to fully exploit the information available on the amplitude of the photon counts [16]. To improve signal-to-noise, PCH was extended to consider longer dwell times in the observation volume [17–19]: molecules then have time to move to regions with different intensities of illumination so that their diffusion affects the data. By considering many dwell-times, these techniques become sensitive to both diffusion and brightness. Yet the PCH methods only approximate the dependence on diffusion, which can lead to errors in the estimation of the higher order moments [11, 20], and any alternatives are computationally intensive [18, 21, 22].

Here we present a method that is not only sensitive to both brightness and diffusion, but also unites FCS, PCH, and moment analysis. The new technique, which we call correlated photon-counting histograms (cPCH), generalizes dual-color PCH [23] so that the two channels being compared can now be shifted in time. Like PCH, the histograms depend on brightness, but how these histograms change from one time point to another depends on diffusion. The histograms can be generated using data either from the same channel or from two different channels, and, although we will focus on single channel measurements, the theoretical expressions derived apply generally.

We also show that cPCH can be applied to the study of images, just as raster image correlation spectroscopy (RICS) extends FCS [24] and spatial intensity distribution analysis (SpIDA) extends PCH [25]. Spatial cPCH can be used to resolve combinations of both immobile and mobile proteins, and like cPCH can resolve different species based on their brightness and diffusion properties. We demonstrate its potential using a simulated system of a fluorescent ligand binding to a receptor with two distinct binding sites.

## Theoretical foundations

### Deriving cPCH

To maintain sensitivity to both the brightness and diffusion of the fluorophores, we need to simultaneously employ information on both the correlation and amplitude of the photon counts. FCS-like methods are sensitive to diffusion through measuring correlations between fluctuations in the fluorescence signal over time; PCH-like methods are sensitive to brightness through analyzing fluctuations in the amplitude of the signal. Our approach is to calculate histograms of the number of photons detected in time bins that occur a time *τ* apart.

We allow each channel to be stimulated by a different laser (the observation volumes in each channel can therefore be of different sizes) and refer to the two channels as channel *A* and channel *B*. Each diffusing molecule has a different brightness profile in each channel, *I*_*A*_(**x**) and *I*_*B*_(**x**), with **x** representing a three dimensional coordinate. We assume that the sampling time *T* is smaller than *τ*_*d*_, the time to diffuse through the observation volume, so that molecules can be considered stationary during each sampling event. For a stationary molecule, the emission and detection of photons in each channel will be uncorrelated (shot noise), and each will follow a Poisson distribution with an average rate that depends upon the fluorophore’s position in the observation volume.

We aim to calculate *p*_*N*_(*n*_*x*_, *n*_*y*_|*τ*), the probability of detecting *n*_*x*_ photons at time 0 and *n*_*y*_ photons at a time *τ* later for a sample containing on average *N* molecules in the observation volume. To begin, we consider the case of a single molecule diffusing in the observation volume [8, 26] and assume that the binning time *T* is much smaller than the diffusion time *τ*_*d*_ of the molecules through the observation volume.

For a single molecule, the probability of interest is *p*_1_(*n*_*x*_, *n*_*y*_|*τ*). As we do not know the position of the molecule, we integrate the probability of detecting *n*_*x*_ photons from a molecule at position **x** and *n*_*y*_ photons at a position **y** over all possible positions:

$$\begin{array}{ccc} p_{1}\left(n_{x},n_{y}|\tau \right) & = & \int p_{1}\left(n_{x},n_{y},x,y|\tau \right)dxdy\\ & = & \int p_{1}\left(n_{y}|y\right)p_{1}\left(n_{x}|x\right)p\left(x,y|\tau \right)dxdy \end{array}$$ (1)

assuming that shot noise is uncorrelated.

The term *p*(**x**, **y**|*τ*) in Eq 1 is an important difference between this equation and the single-molecule probability functions for PCH and dual-color PCH [26]. This term defines the probability of being in position **x** at time 0 and position **y** at time *τ*. For free diffusion (without boundaries) and if *D* is the diffusion coefficient of the fluorophore, then

$$\begin{array}{ccc} p\left(x,y|\tau \right) & = & p(y|x,\tau )p(x|\tau )\\ & = & \frac{1}{{\left(4\pi D\tau \right)}^{\frac{3}{2}}}e^{-\frac{{\left(y-x\right)}^{2}}{4D\tau }}\frac{1}{V} \end{array}$$ (2)

where the probability *p*(**x**|*τ*) is the probability density of finding the molecule at **x** at time *τ* and is 1/*V*, where *V* is the experimental volume (which is larger than the observational volume *V*_AB_).

Rather than performing the integration in Eq 1, we instead find a solution using Taylor series. If the probability of obtaining *n* photon counts from any given position depends only on the illumination intensity at that position, *I*(**x**), then

$$\begin{array}{c} p_{1}\left(n|x\right)=\frac{I{\left(x\right)}^{n}e^{-I(x)}}{n!}. \end{array}$$ (3)

Eq 3 implies that Eq 1 contains exponentials of *I*(**x**) + *I*(**y**), which we Taylor expand and then use the binomial theorem to expand further into terms in *I*(**x**) and in *I*(**y**). Reversing the order of integration and summation gives

$$p_{1}\left(n_{x},n_{y}|\tau \right)=\frac{1}{V}\sum_{k=0}^{\infty }\frac{{\left(-1\right)}^{k}}{n_{x}!n_{y}!k!}\sum_{m=0}^{k}(\begin{array}{c} k\\ m \end{array})\int {I_{A}\left(x\right)}^{n_{x}+k-m}{I_{B}\left(y\right)}^{n_{y}+m}p\left(x,y|\tau \right)dxdy$$ (4)

Without the diffusion term, *p*(**x**, **y**|*τ*), the integral in Eq 4 gives the moments of the brightness profile. Eq 4 therefore reduces to a series solution for the single-molecule case of dual-color PCH. With the diffusion term, the integral gives a higher-order two-point correlation function, *G*_*m*,*n*_(*τ*):

$$\begin{array}{c} G_{m,n}\left(\tau \right)=\frac{1}{V_{\text{AB}}}\int {I_{A}\left(x\right)}^{m}{I_{B}\left(y\right)}^{n}p\left(x,y|\tau \right)dxdy \end{array}$$ (5)

where *V*_AB_ is the volume of the observation region (found from the point spread function). Consequently, Eq 4 can be written as

$$\begin{array}{c} p_{1}\left(n_{x},n_{y}|\tau \right)=\frac{V_{\text{AB}}}{V}\sum_{k=0}^{\infty }\frac{{\left(-1\right)}^{k}}{n_{x}!n_{y}!k!}\sum_{m=0}^{k}(\begin{array}{c} k\\ m \end{array})G_{n_{x}+k-m,n_{y}+m}\left(\tau \right). \end{array}$$ (6)

To find explicit expressions for the two-point correlation functions (Eq 5), we use a Gaussian profile for the intensity of illumination [16, 18, 27]:

$$\begin{array}{c} I_{A}\left(x\right)=\epsilon_{A}\exp[-\frac{2(x_{x}^{2}+x_{y}^{2})}{r_{A}^{2}}-\frac{2x_{z}^{2}}{z_{A}^{2}}] \end{array}$$ (7)

where *r*_*A*_ and *z*_*A*_ denote the radial and axial beam waist parameters, and *ϵ*_*A*_ is the constant brightness of the single molecule in channel *A* in units of photon counts/bin (of duration *T* seconds). A similar expression holds for *I*_*B*_(**x**). Eq 7 implies that the two-channel observation volume is (as in fluorescence cross-correlation spectroscopy [28]):

$$\begin{array}{c} V_{\text{AB}}={\left(\frac{\pi }{2}\right)}^{3/2}(\frac{{r_{A}}^{2}+{r_{B}}^{2}}{2}){\left(\frac{{z_{A}}^{2}+{z_{B}}^{2}}{2}\right)}^{1/2}. \end{array}$$ (8)

Independently treating each spatial dimension in Eq 5 gives

$$\begin{array}{c} G_{m,n}\left(\tau \right)={\epsilon_{A}}^{m}{\epsilon_{B}}^{n}\gamma_{m,n}\kappa_{m,n}\left(\tau ,D\right) \end{array}$$ (9)

where *κ*_*m*,*n*_(*τ*, *D*) is

$$\begin{array}{c} \kappa_{m,n}\left(\tau ,D\right)={\left[1+\frac{8mnD\tau }{n{r_{A}}^{2}+m{r_{B}}^{2}}\right]}^{-1}{\left[1+\frac{8mnD\tau }{n{z_{A}}^{2}+m{z_{B}}^{2}}\right]}^{-\frac{1}{2}}. \end{array}$$ (10)

The shape factors of the observation volume are defined as

$$\begin{array}{c} \gamma_{m,n}=\frac{\int {\left(\frac{I_{A}\left(x\right)}{\epsilon_{A}}\right)}^{m}{\left(\frac{I_{B}\left(x\right)}{\epsilon_{B}}\right)}^{n}dx}{V_{\text{AB}}} \end{array}$$ (11)

and obey

$$\begin{array}{c} \gamma_{m,n}=4{\left[(\frac{m}{{r_{A}}^{2}}+\frac{n}{{r_{B}}^{2}})({r_{A}}^{2}+{r_{B}}^{2}){\left(\frac{2m}{{z_{A}}^{2}}+\frac{2n}{{z_{B}}^{2}}\right)}^{1/2}{\left({z_{A}}^{2}+{z_{B}}^{2}\right)}^{1/2}\right]}^{-1} \end{array}$$ (12)

for the Gaussian intensity profile.

If the two channels have the same observation volume (*A* = *B*), Eq 10 becomes the correlation function derived by Melnykov *et al*. [16]. The shape factors simplify to *γ*_*m*,*n*_ = *γ*_*m*+*n*_ and become the shape factors for a single channel ($\gamma_{n}=n^{-\frac{3}{2}}$ for a Gaussian intensity profile).

#### Extending to *N* molecules

We have considered the probability of detecting photons from single molecules, but the molecules of interest will diffuse in and out of the laser’s observation volume, and the actual number of molecules in the observation volume at any given time will fluctuate around the average value *N*. These fluctuations are also driven by a Poisson process and so the probability of detecting *n*_*x*_ photons from channel *A* and *n*_*y*_ photons from channel *B* will be given by a compound Poisson distribution.

First, we briefly discuss a technical point. The fraction *V*_AB_/*V* in Eq 6 is the ratio of the observation volume to the larger experimental volume, but can be set to unity when we consider more than one molecule in the observation volume because this ratio then cancels in the generating function [29]. Although we should consider the number of molecules in the larger experimental volume, *N*_*v*_, we will assume *V*_AB_ = *V* and employ *N* = *N*_*v*_ throughout because a constant concentration of molecules implies *N*_*v*_/*V* = *N*/*V*_AB_.

The probability of detecting *n*_*x*_ photons in channel *A* and *n*_*y*_ photons in channel *B* at a time *τ* later when there are *N* molecules on average in the observation volume can be found by marginalizing the probability of detecting those photons given there are *m* molecules exactly in the observation volume multiplied by the probability of detecting these *m* molecules if there are *N* in the observation volume on average:

$$\begin{array}{c} p_{N}\left(n_{x},n_{y}|\tau \right)=\sum_{m=0}^{\infty }p\left(n_{x},n_{y}|\tau ,m\right)p\left(m|N\right) \end{array}$$ (13)

where *p*(*m*|*N*) is given by a Poisson distribution,

$$\begin{array}{c} p\left(m|N\right)=\frac{e^{-N}N^{m}}{m!}. \end{array}$$ (14)

The probability *p*(*n*_*x*_, *n*_*y*_|*τ*, *m*) is given by the *m*-fold convolution of the single molecule probabilities. The probability density of a sum of independent variables (*n*_*x*_ and *n*_*y*_ are both sums over the photons emitted by each molecule) is the convolution of the individual probability densities [30] (these densities determine the individual $n_{x}^{\prime }$ and $n_{y}^{\prime }$ emitted by each molecule). Therefore we have

$$\begin{array}{c} p\left(n_{x},n_{y}|\tau ,m\right)=p_{1}\left(n_{x},n_{y}|\tau \right)*p_{1}\left(n_{x},n_{y}|\tau \right)*\ldots *p_{1}\left(n_{x},n_{y}|\tau \right)\;(m\;\text{times}). \end{array}$$ (15)

To proceed, we use the bivariate probability generating function given by:

$$\begin{array}{c} g_{N}\left(t,s|\tau \right)=\sum_{n_{x}=0}^{\infty }\sum_{n_{y}=0}^{\infty }t^{n_{x}}s^{n_{y}}p_{N}\left(n_{x},n_{y}|\tau \right) \end{array}$$ (16)

and note that the *m*-fold convolution of a probability distribution is equivalent to raising its generating function to the *m*’th power [30]. If *g*_1_(*t*, *s*|*τ*) is the generating function for the single-molecule probability distribution, we can write

$$\begin{array}{ccc} g_{N}\left(t,s|\tau \right) & = & \sum_{n_{x}=0}^{\infty }\sum_{n_{y}=0}^{\infty }t^{n_{x}}s^{n_{y}}\sum_{m=0}^{\infty }p\left(n_{x},n_{y}|\tau ,m\right)p\left(m|N\right)\\ & = & \sum_{m=0}^{\infty }{g_{1}\left(t,s|\tau \right)}^{m}p\left(m|N\right)\\ & = & \sum_{m=0}^{\infty }{g_{1}\left(t,s|\tau \right)}^{m}\frac{e^{-N}N^{m}}{m!}\\ & = & \exp[N(g_{1}\left(t,s|\tau \right)-1)]. \end{array}$$ (17)

We have an exponential generating function in Eq 17, and we will show that this property translates into a sum of the different single molecule generating functions, weighted by the concentration of each species, because the generating function of a sum of independent random variables is the product of their individual generating functions [30]. If there are *w* independent, fluorescent molecular species in the observation volume (which do not have substantial interactions over the time scale of the experiment), the generating function can be written as

$$\begin{array}{c} g_{N_{w}}\left(t,s|\tau \right)=\exp[\sum_{q=1}^{w}N_{q}(g_{1}^{\left(q\right)}\left(t,s|\tau \right)-1)]. \end{array}$$ (18)

For better comparison with experiments, we also include a constant (Poisson) background noise in each channel. This noise has an average of λ_*A*_ counts per bin time *T* in channel *A* and λ_*B*_ counts per bin time *T* in channel *B*. The generating function is then

$$\begin{array}{c} g_{N_{w}}\left(t,s|\lambda_{A},\lambda_{B},\tau \right)=\exp([\sum_{q=1}^{w}N_{q}(g_{1}^{\left(q\right)}\left(t,s|\tau \right)-1)]+\lambda_{A}\left(t-1\right)+\lambda_{B}\left(s-1\right)). \end{array}$$ (19)

Although later we will derive a recursion relation for the cPCH probability distribution from Eq 19, it is often more efficient to use the Fourier transform to evaluate the generating functions (replacing *t* and *s* in Eq 16 by e^*it*′^ and e^*is*′^ [30]) and directly calculate the cPCH distributions using the inverse Fourier transform, $F^{-1}$:

$$\begin{array}{cc} p_{N_{w}}\left(n_{x},n_{y}|\lambda_{A},\lambda_{B},\tau \right) & =F^{-1}(\exp([\sum_{q=1}^{w}N_{q}(g_{1}^{\left(q\right)}\left(t^{\prime },s^{\prime }|\tau \right)-1)]+\\ & \;\log(F(p(n_{x},n_{y}|\lambda_{A},\lambda_{B}))))) \end{array}$$ (20)

where the second term describes the background noise. The generating functions for each single molecule probability distribution, $g_{1}^{\left(q\right)}\left(t^{\prime },s^{\prime }|\tau \right)$, are calculated using the Fourier transform of the single molecule probability distributions *p*_1_(*n*_*x*_, *n*_*y*_|*τ*). The joint probability in the background noise is

$$\begin{array}{c} p\left(n_{x},n_{y}|\lambda_{A},\lambda_{B}\right)=\text{Poisson}\left(n_{x},\lambda_{A}\right)\text{Poisson}\left(n_{y},\lambda_{B}\right) \end{array}$$ (21)

and, using the properties of the discrete Fourier transform,

$$\begin{array}{c} \log\left(F(p(n_{x},n_{y}|\lambda_{A},\lambda_{B}))\right)=\lambda_{a}[e^{\left(-it^{\prime }\frac{2\pi }{L_{t^{\prime }}}\right)}-1]+\lambda_{b}[e^{\left(-is^{\prime }\frac{2\pi }{L_{s^{\prime }}}\right)}-1] \end{array}$$ (22)

where *L*_*s*′_ and *L*_*t*′_ are the numbers of elements in *s*′ and *t*′. We note that different species are included in Eq 20 by summing their single-molecule generating functions weighted by their concentrations rather than the successive convolutions of the PCH approach [8, 26].

#### Recursive solution

The probability generating function can also be used to derive the probabilities. By definition [30]

$$\begin{array}{c} p\left(n_{x},n_{y}\right)=\lim_{s\to 0}\lim_{t\to 0}\frac{1}{n_{x}!n_{y}!}\frac{d^{n_{x}}}{ds^{n_{x}}}\frac{d^{n_{y}}}{dt^{n_{y}}}g\left(s,t\right) \end{array}$$ (23)

and exploiting the Leibniz rule for differentiation (for both *s* and *t*)

$$\begin{array}{c} \frac{d^{n}}{dt^{n}}f\left(t\right)g\left(t\right)=\sum_{k=0}^{n}(\begin{array}{c} n\\ k \end{array})\frac{d^{n-k}}{dt^{n-k}}f\left(t\right)\frac{d^{k}}{dt^{k}}g\left(t\right), \end{array}$$ (24)

and writing $p_{1_{i}}\left(n_{1},n_{2}|\tau \right)$ for the single-molecule probability, Eq 1, for species *i*, we find the following recursion for the cPCH probability distribution:

- for *n*_*x*_ > 0 and *n*_*y*_ > 0:
  $$\begin{array}{ccc} p_{N_{w}}\left(n_{x},n_{y}|\lambda_{A},\lambda_{B},\tau \right) & = & \frac{\lambda_{A}}{n_{x}}p_{N_{w}}\left(n_{x}-1,n_{y}|\lambda_{A},\lambda_{B},\tau \right)\\ & & +\sum_{z=0}^{n_{y}}\sum_{q=1}^{n_{x}}\frac{q}{n_{x}}p_{N_{w}}\left(n_{x}-q,n_{y}-z|\tau ,\lambda_{A},\lambda_{B}\right)\\ & & \times \sum_{i=1}^{w}N_{i}p_{1_{i}}\left(q,z|\tau \right) \end{array}$$ (25)
- for *n*_*x*_ > 0 and *n*_*y*_ = 0:
  $$\begin{array}{ccc} p_{N_{w}}\left(n_{x},n_{y}|\lambda_{A},\lambda_{B},\tau \right) & = & \frac{\lambda_{A}}{n_{x}}p_{N_{w}}\left(n_{x}-1,0|\lambda_{A},\lambda_{B},\tau \right)\\ & & +\sum_{q=1}^{n_{x}}\frac{q}{n_{x}}p_{N_{w}}\left(n_{x}-k,0|\lambda_{A},\lambda_{B},\tau \right)\\ & & \times \sum_{i=1}^{w}N_{i}p_{1_{i}}\left(q,0|\tau \right) \end{array}$$ (26)
- for *n*_*x*_ = 0 and *n*_*y*_ > 0:
  $$\begin{array}{ccc} p_{N_{w}}\left(n_{x},n_{y}|\lambda_{A},\lambda_{B},\tau \right) & = & \frac{\lambda_{B}}{n_{y}}p_{N_{w}}\left(0,n_{y}-1|\lambda_{A},\lambda_{B},\tau \right)\\ & & +\sum_{q=1}^{n_{y}}\frac{q}{n_{y}}p_{N_{w}}\left(0,n_{y}-k|\lambda_{A},\lambda_{B},\tau \right)\\ & & \times \sum_{i=1}^{w}N_{i}p_{1_{i}}\left(0,q|\tau \right) \end{array}$$ (27)
- for *n*_*x*_ = *n*_*y*_ = 0:
  $$p_{N_{w}}(n_{x},n_{y}|\lambda_{A},\lambda_{B},\tau )=\text{exp}\left[-\sum_{i=1}^{w}N_{i}(1-p_{1_{i}}(0,0|\tau ))-\lambda_{A}-\lambda_{B}\right].$$ (28)

To calculate the recursion we start at *n*_*x*_ = *n*_*y*_ = 0, followed by *n*_*x*_ = 0 and *n*_*y*_ = 1, and then by *n*_*x*_ = 1 and *n*_*y*_ = 0 before continuing with successively higher values of *n*_*x*_ and *n*_*y*_.

Eq 25 is also applicable to dual color PCH if *τ* = 0. Analytical expressions for single-channel PCH can be derived similarly [31, 32].

### Moments

Similar to PCH [11, 27], the moments of the cPCH distributions efficiently summarize the relevant information. The moments are easier to compute than the full distribution, and we can express all of the moments, and even the single-molecule probability distributions, in terms of the factorial moments of the single-molecule distributions.

For moments of order *m* for *n*_*x*_ and *n* for *n*_*y*_, we use the notation: *M*_*m*,*n*_ for the raw moment (about zero); (*M*)_*m*,*n*_ for the factorial moment; *K*_*m*,*n*_ for the cumulant; and (*K*)_*m*,*n*_ for the factorial cumulant. All these quantities can be found through generating functions. As well as the probability generating function, Eq 16, the moment generating function is required:

$$\begin{array}{c} g^{\left(M\right)}\left(t,s\right)=\sum_{m=0}^{\infty }\sum_{n=0}^{\infty }e^{tm}e^{sn}p\left(m,n\right). \end{array}$$ (29)

The raw moments, *M*_*m*,*n*_, and cumulants, *K*_*m*,*n*_, are found from this moment generating function. Differentiating *g*^(*M*)^(*t*, *s*), *m* times with respect to *t* and *n* times with respect to *s*, and then setting *s* = *t* = 0 gives the raw moments; differentiating the logarithm of *g*^(*M*)^(*t*, *s*) and setting *s* = *t* = 0 gives the cumulants. The factorial moments, (*M*)_*m*,*n*_, and cumulants, (*K*)_*m*,*n*_, are found from the probability generating function, Eq 16. Differentiating *g*(*t*, *s*), and setting *s* = *t* = 1 gives the factorial moments; differentiating the logarithm of *g*(*t*, *s*) and setting *s* = *t* = 1 gives the factorial cumulants.

#### Factorial moments for a single molecule

Using Eq 3 in Eq 1 and the definition of the probability generating function, Eq 16, we find that *g*_1_(*t*, *s*|*τ*) is

$$\begin{array}{c} g_{1}\left(t,s|\tau \right)=\frac{1}{V}\int \int e^{I_{A}\left(x\right)\left(t-1\right)+I_{B}\left(y\right)\left(s-1\right)}p\left(x,y|\tau \right)dxdy \end{array}$$ (30)

after performing the sums over *n*_*x*_ and *n*_*y*_. Differentiating Eq 30, we find that the factorial moments of the single molecule distribution are

$$\begin{array}{c} {\left(M\right)}_{m,n}\left(\tau \right)=\frac{V_{\text{AB}}}{V}G_{m,n}\left(\tau \right) \end{array}$$ (31)

with *G*_*m*,*n*_(*τ*) being given by Eq 9.

We note that comparing Eq 6 and Eq 31 allows the single-molecule probability distributions to be expressed as an infinite sum over the factorial moments:

$$\begin{array}{c} p_{1}\left(n_{x},n_{y}|\tau \right)=\sum_{k=0}^{\infty }\frac{{\left(-1\right)}^{k}}{n_{x}!n_{y}!k!}\sum_{m=0}^{k}(\begin{array}{c} k\\ m \end{array}){\left(M\right)}_{n_{x}+k-m,n_{y}+m}\left(\tau \right). \end{array}$$ (32)

#### Factorial cumulants for *N* molecules

For *N* molecules, the factorial cumulants provide the simplest expressions:

$$\begin{array}{c} {\left(K\right)}_{m,n}\left(\tau \right)=NG_{m,n}\left(\tau \right). \end{array}$$ (33)

The factorial cumulants for the *N* molecule case are therefore the higher-order two-point correlation function, Eq 5, multiplied by the average number of molecules *N*.

We can extend these results to *w* different species by noting that the logarithm of the generating function for the multiple molecule case, Eq 18, is the sum of the individual generating functions weighted by their concentrations. We can therefore sum the factorial cumulants of each individual species. For better comparison with experiment, we include a Poisson background noise of λ_*A*_ counts per bin in channel *A* and λ_*B*_ counts per bin in channel *B* by noting that only the first factorial cumulant of a Poisson distribution is non-zero (and is identical to the mean of the distribution). We find

$$\begin{array}{ccc} {\left(K^{\left(w\right)}\right)}_{1,0} & = & \lambda_{A}+\sum_{i=1}^{w}N_{i}\epsilon_{A_{i}}\gamma_{1,0}\\ {\left(K^{\left(w\right)}\right)}_{0,1} & = & \lambda_{B}+\sum_{i=1}^{w}N_{i}\epsilon_{B_{i}}\gamma_{0,1} \end{array}$$ (34)

and

$$\begin{array}{c} {\left(K^{\left(w\right)}\right)}_{m,n}=\gamma_{m,n}\sum_{i=1}^{w}N_{i}{\epsilon_{A_{i}}}^{m}{\epsilon_{B_{i}}}^{n}\kappa_{m,n}\left(\tau ,D_{i}\right) \end{array}$$ (35)

for *m*, *n* > 1. Here *κ*_*m*,*n*_ and *γ*_*m*,*n*_ are defined in Eqs 10 and 11.

Further, we can derive recurrence relations to convert the factorial cumulants into cumulants and raw moments, which is necessary for comparing theory with experimental results (Sec. S1).

### Moments of moments

The method of moments of moments is used to estimate the experimental uncertainty of higher order factorial cumulants [11]. Rather than using *k*-statistics [27], we note that the variance of a random variable can be expressed as a Taylor series of its raw moments [33]. To first order in *N*_*d*_^−1^ with *N*_*d*_ being the number of data points (typically on the order of 10^6^), we can write the variance of either the cumulant or factorial cumulant of interest, *X*_*p*,*r*_, as

$$\begin{array}{c} \text{Var}\left[X_{p,r}\right]sum_{x,u=0}^{p}\sum_{y,v=0}^{r}\frac{\partial X_{p,r}}{\partial M_{x,y}}\frac{\partial X_{p,r}}{\partial M_{u,v}}\text{Cov}\left[M_{x,y},M_{u,v}\right] \end{array}$$ (36)

where Cov[*M*_*x*,*y*_, *M*_*u*,*v*_] is the covariance of the two raw moments *M*_*x*,*y*_ and *M*_*u*,*v*_ and is given by [33]

$$\begin{array}{c} \text{Cov}\left[M_{x,y},M_{u,v}\right]=\frac{1}{N_{d}}\left(M_{x+u,y+v}-M_{x,y}M_{u,v}\right). \end{array}$$ (37)

Using the recurrence relations in Sec. S1, we can express the factorial cumulants in terms of the raw moments and so evaluate the derivatives in Eq 36. We provide examples of variances of the factorial cumulants in Sec. S9.

Finally, we note that we have assumed that the photon counts are independent of each other, which is not true in practice, and a correction is required for the variance of the first factorial cumulant [18].

### Obtaining previous methods in fluorescence fluctuation spectroscopy

#### FCS

We can calculate an FCS curve from the moments of the cPCH distribution. The normalized autocorrelation function of FCS is:

$$\begin{array}{c} G_{\text{FCS}}\left(\tau \right)=\frac{E[n_{x}\left(t\right)n_{y}\left(t+\tau \right)]}{E\left[n_{x}\left(t\right)\right]E\left[n_{y}\left(t+\tau \right)\right]} \end{array}$$ (38)

or

$$\begin{array}{c} G_{\text{FCS}}\left(\tau \right)=\frac{M_{1,1}\left(\tau \right)}{M_{1,0}\left(\tau \right)M_{0,1}\left(\tau \right)} \end{array}$$ (39)

using the definitions of the raw moments of the cPCH distribution. Via the relations in Sec. S1, we can also express Eq 38 in terms of the factorial cumulants:

$$\begin{array}{c} G_{\text{FCS}}\left(\tau \right)=1+\frac{{\left(K\right)}_{1,1}\left(\tau \right)}{{\left(K\right)}_{1,0}\left(\tau \right){\left(K\right)}_{0,1}\left(\tau \right)}. \end{array}$$ (40)

Fitting FCS curves requires the error at each point of the curve [34]. With cPCH, not only do the analytical solutions to the factorial cumulants provide expressions for fitting, but also the the variances calculated by the moments of moments technique estimate the errors. In addition, we can include dead-time and afterpulsing effects in the cPCH distribution and so also in the FCS curve [35].

Care is needed when *τ* = 0. Then *n*_*x*_ = *n*_*y*_ by definition because we are comparing each time bin with itself: the empirical probability distribution *p*(*n*_*x*_, *n*_*y*_|*τ* = 0) will be zero everywhere except along the diagonal where *n*_*x*_ = *n*_*y*_, and the data are one (not two) dimensional.

For FCS, we therefore define

$$\begin{array}{c} M_{1,1}\left(\tau \right)=\{\begin{array}{cc} {\left(K\right)}_{1,1}\left(\tau \right)+{\left(K\right)}_{0,1}{\left(K\right)}_{1,0} & \tau >0\\ {\left(K\right)}_{1}+{{\left(K\right)}_{1}}^{2}+{\left(K\right)}_{2} & \tau =0 \end{array} \end{array}$$ (41)

replacing the relationship for the joint moment by the equivalent relationship for the second moment of a one dimensional distribution when *τ* = 0.

#### PCH

We can obtain a single-channel PCH at any *τ* by summing over all counts on one of the two axes of the cPCH distribution, *p*_*N*_(*n*_*x*_, *n*_*y*_|*τ*), because *p*_*N*_(*n*_*x*_) = *p*_*N*_(*n*_*x*_|*τ*) and $p_{N}\left(n_{x}|\tau \right)=\sum_{n_{y}}p_{N}\left(n_{x},n_{y}|\tau \right)$.

For example, using the single-molecule probability distribution *p*_1_(*n*_*x*_, *n*_*y*_|*τ*) (Eq 3 in Eq 1):

$$\begin{array}{ccc} p_{1}\left(n_{x}\right) & = & \sum_{n_{y}=0}^{\infty }p_{1}\left(n_{x},n_{y}|\tau \right)\\ & = & \sum_{n_{y}=0}^{\infty }\frac{1}{V}\int \frac{{I_{A}\left(x\right)}^{n_{x}}e^{-I_{A}\left(x\right)}}{n_{x}!}\frac{{I_{B}\left(y\right)}^{n_{y}}e^{-I_{B}\left(y\right)}}{n_{y}!}\frac{1}{{\left(4\pi D\tau \right)}^{\frac{3}{2}}}e^{-\frac{{\left(y-x\right)}^{2}}{4D\tau }}dxdy\\ & = & \frac{1}{V}\int \frac{{I_{A}\left(x\right)}^{n_{x}}e^{-I_{A}\left(x\right)}}{n_{x}!}dx \end{array}$$ (42)

carrying out first the sum over *n*_*y*_ and then the integral over **y**. This integral over **x** is the definition of the single-molecule probability distribution used in PCH [8].

Obtaining a PCH curve at any *τ* is useful: in cPCH, it can be beneficial to use larger bin sizes *T* as *τ* increases to increase the signal-to-noise ratio, a common practice in FCS. Using Eq 42, it is therefore straightforward to obtain PCH curves at different bin sizes, *p*_1_(*n*_*x*_|*T*), increasing the effective counts/bin to improve the signal statistics, following FIMDA [17, 19]. Using several different *T* also provides additional constraints on the brightness in the fitting routines. As in FIMDA, the effect of molecular diffusion during longer bin times on the cPCH distributions must also be included and will be discussed below. To calculate the theoretical *p*_1_(*n*_*x*_|*T*), we can take diffusion into consideration during the bin time *T* by using Eq 51 for the factorial moments in the single molecule series solution (Eq 32).

#### FCA

We can obtain the fluorescence cumulants used in FCA [11] by setting *m* (or *n*) to zero in Eq 33:

$$\begin{array}{c} {\left(K\right)}_{n}\left(\tau \right)=N{\epsilon_{a}}^{n}\gamma_{n,0}. \end{array}$$ (43)

We note that the definition of *γ*_*m*,*n*_ in Eq 12 includes any differences in the size of volume between the two channels so that (*K*)_*n*,0_(*τ*) will yield the same numerical value as (*K*)_*n*_ from FCA.

#### Dual-color PCH

The dual-color PCH distribution is the dual-color cPCH distribution with a zero time-shift:

$$\begin{array}{c} p_{N}\left(n_{x},n_{y}\right)=p_{N}\left(n_{x},n_{y}|\tau =0\right). \end{array}$$ (44)

### Diffusion effects at large bin sizes

We have assumed that the binning time *T* is smaller than the diffusion time *τ*_*d*_ of the molecules, but, as *T* increases towards *τ*_*d*_, molecules can no longer be considered stationary in the observation volume and will experience a range of intensities, *I*(x), changing the statistics of the photon counts.

#### FIMDA and TIFCA

To allow larger *T*, we follow Fluorescence Intensity Multiple Distribution Analysis (FIMDA) [17] and introduce a second order binning function *B*_2_(*T*, *τ*_*d*_) so that the factorial cumulants have the same form as Eqs 33 and 43, but with effective brightness and concentration parameters. These effective parameters are:

$$\begin{array}{ccc} \epsilon^{*}\left(T\right)=\epsilon_{o}TB_{2}\left(T,\tau_{d}\right) & ; & N^{*}\left(T\right)=\frac{N}{B_{2}\left(T,\tau_{d}\right)} \end{array}$$ (45)

where *ϵ*_*o*_ is the count rate of the fluorescent molecule per second rather than per bin time *T*, and *B*_2_(*T*, *τ*_*d*_) is [17]:

$$\begin{array}{ccc} B_{2}\left(T,\tau_{d}\right) & = & \frac{4}{\alpha^{2}\beta }[\frac{\beta (1+\alpha )}{\sqrt{1-\beta }}\text{atanh}(\sqrt{1-\beta }\frac{\left(\sqrt{1+\beta \alpha }-1\right)}{\left(\beta +\sqrt{1+\beta \alpha }-1\right)})\\ & & +1-\sqrt{1+\beta \alpha }] \end{array}$$ (46)

assuming a 3D Gaussian observation volume and with *β* = (*r*_*a*_/*z*_*a*_)^2^ and *α* = *T*/*τ*_*d*_.

Moment analysis was also extended to include binning effects using Time Integrated Factorial Cumulant Analysis (TIFCA) [18]. All factorial cumulants above first-order are weighted by a binning function of similar order, i.e.,

$$\begin{array}{cc} {\left(K\right)}_{n}\left(T\right)=N{\left(\epsilon_{o}T\right)}^{n}B_{n}\left(T,\tau_{d}\right)\gamma_{n} & (\text{TIFCA}). \end{array}$$ (47)

which should be compared with Eq 43. Although the higher order binning functions in TIFCA are exact, rather than the approximations used in FIMDA, binning functions with *n* > 2 require more complex numerical integration.

By comparing FIMDA’s approximation with the TIFCA results, we can show that FIMDA’s approximation for the higher order binning functions is

$$\begin{array}{c} B_{n}\left(T,\tau_{d}\right)\approx {B_{2}\left(T,\tau_{d}\right)}^{n-1}. \end{array}$$ (48)

Replacing the concentration and brightness parameters in the factorial cumulant expressions (Eq 43) with FIMDA’s effective concentration parameters, we find that

$$\begin{array}{ccc} {\left(K\right)}_{n}\left(T\right) & = & N^{*}\left(T\right){\left(\epsilon^{*}\left(T\right)\right)}^{n}\gamma_{n}\\ & = & N{\left(\epsilon_{o}T\right)}^{n}{B_{2}\left(T,\tau_{d}\right)}^{n-1}\gamma_{n}\;(\text{FIMDA}). \end{array}$$ (49)

from which follows the approximation in Eq 48.

#### cPCH

Deriving a general equation for *B*_*m*,*n*_(*T*, *τ*_*d*_) is challenging, and we instead use FIMDA’s approximation (Eq 48). From Eq 32, the cPCH probability can be expressed as an infinite series of the factorial moments (*M*)_*m*,*n*_(*τ*, *T*).

We separately consider the order for each channel: the first channel is of order *m* and the second of order *n*, and binning is only required if the order of a given channel is either 2 or higher. Each moment will then be scaled by its binning function *B*_*m*,*n*_(*T*, *τ*_*d*_). Although dual color PCH [26] and TIFCA [27] (equivalent to cPCH when *τ* = 0) use a second order binning function for *K*_11_(*T*), we have found that this approach applied to single color distributions matches neither simulated nor experimental data for *τ* > 0.

For cPCH, we thus approximate the binning functions using Eq 48:

$$\begin{array}{c} B_{m,n}\left(T,\tau_{d}\right)\approx \{\begin{array}{cc} B_{2}{\left(T,\tau_{A}\right)}^{m-1}, & n=0\\ B_{2}{\left(T,\tau_{B}\right)}^{n-1}, & m=0\\ B_{2}{\left(T,\tau_{A}\right)}^{m-1}B_{2}{\left(T,\tau_{B}\right)}^{n-1}, & m>0\;\text{and}\;n>0 \end{array} \end{array}$$ (50)

where the diffusion time for channel A, *τ*_*A*_, is not necessarily the same as the diffusion time for channel *B*, *τ*_*B*_, because the two observation volumes may be of different sizes.

Using Eqs 31 and 9, we can now define the factorial moments in Eq 32 as

$$\begin{array}{c} {\left(M\right)}_{m,n}\left(\tau ,T\right)=\frac{V_{\text{AB}}}{V}{\left(\epsilon_{a_{o}}T\right)}^{m}{\left(\epsilon_{b_{o}}T\right)}^{n}\kappa_{m,n}\left(\tau ,\tau_{d}\right)B_{m,n}\left(T,\tau_{d}\right)\gamma_{m,n} \end{array}$$ (51)

to include binning effects. Similarly, the factorial cumulants for a single species become

$$\begin{array}{c} {\left(K\right)}_{m,n}\left(\tau ,T\right)=N{\left(\epsilon_{a_{o}}T\right)}^{m}{\left(\epsilon_{b_{o}}T\right)}^{n}\kappa_{m,n}\left(\tau ,\tau_{d}\right)B_{m,n}\left(T,\tau_{d}\right)\gamma_{m,n} \end{array}$$ (52)

from Eqs 9 and 33.

### Extending to flow and images

We can extend cPCH to the study of images. Correlations between different regions of an image should only occur if the same molecule has visited both regions. We focus on detectors run in photon-counting mode and raster-scanned images. We therefore need to include the probability of detecting the same molecule at two different positions at two different times. This movement of the scanning laser is analogous to a directed flow and, similar to raster-scanned image correlation spectroscopy [24, 36], we include a flow term in the diffusion part of the correlation function (Eq 9). This change is the only one needed.

If there is a flow *v*_*x*_ in the *x*-direction and *v*_*y*_ in the *y*-direction, we can write the correlation function as

$$\begin{array}{c} G_{m,n}\left(\tau \right)=\frac{1}{V_{\text{AB}}}\int {I_{A}\left(x\right)}^{m}{I_{B}\left(y\right)}^{n}\frac{1}{{\left(4\pi D\tau \right)}^{\frac{3}{2}}}e^{-\frac{{\left(y-x-v\tau \right)}^{2}}{4D\tau }}dxdy \end{array}$$ (53)

or

$$\begin{array}{c} G_{m,n}\left(\tau \right)=\frac{V_{\text{AB}}}{V}{\epsilon_{a}}^{m}{\epsilon_{b}}^{n}\gamma_{m,n}S_{m,n}\left(\tau ,\tau_{d}\right)\kappa_{m,n}\left(\tau ,D\right) \end{array}$$ (54)

where *κ*_*m*,*n*_(*τ*, *D*) is the diffusion term of Eq 10 and *S*_*m*,*n*_(*τ*, *D*) describes the contribution from flow:

$$\begin{array}{c} S_{m,n}\left(\tau ,D\right)=\exp[-\frac{2mn\tau^{2}\left(v_{x}^{2}+v_{y}^{2}\right)}{\left(n{r_{A}}^{2}+m{r_{B}}^{2})(1+\frac{8mn\tau D}{n{r_{A}}^{2}+m{r_{B}}^{2}}\right)}] \end{array}$$ (55)

assuming a 3D-Gaussian observation volume in each of the two channels.

The times *τ* that we can consider depend on the time taken by the laser to travel from one pixel to another. To translate the distance between pixels into a time [24], we use the line-retracing time, *τ*_*L*_, the pixel dwell time, *τ*_*p*_, the pixel size, *δ*_*x*_ by *δ*_*y*_, and the number of pixels in a line, *L*_*y*_, so that *v*_*x*_*τ* = *d*_*x*_*δ*_*x*_ and *v*_*y*_*τ* = *d*_*y*_*δ*_*y*_ in Eq 55 for a pixel located *d*_*x*_ pixels away in the horizontal direction and *d*_*y*_ pixels away in the vertical direction. The time for the laser to reach the position *d*_*x*_ in the current line is *τ*_*p*_*d*_*x*_; the time for the laser to reach the position *d*_*y*_ by scanning the previous lines is *τ*_*p*_*L*_*y*_*d*_*y*_; and *τ*_*L*_*d*_*y*_ is the corresponding line-retracing time. Consequently, *τ* in Eq 55 becomes *τ*_*p*_(*d*_*x*_ + *L*_*y*_*d*_*y*_) + *τ*_*L*_*d*_*y*_.

With Eq 53 for *G*_*m*,*n*_(*τ*), we can calculate the probabilities *p*_*N*_(*n*_*A*_, *n*_*B*_|*d*_*x*_, *d*_*y*_) and the associated moments as before (for example, using Eq 6). We note that for images of cell membranes, 2D diffusion is more appropriate, and Eqs 8, 10 and 11 should be replaced by their 2D equivalents.

### Triplet states and detector dead-time and afterpulsing

As with other methods in fluorescence fluctuation spectroscopy, cPCH can be modified to correct for non-ideal behaviour from fluorophores and detectors. We correct for triplet states and fluorophore blinking following FCS [37] and FIMDA [17] (Sec. S6) and for detector dead-time following Melnykov and Hall [16] (Sec. S7). For afterpulsing by the detector, we build on previous work [38], but develop corrections that include the probability of detecting an afterpulse at a time *τ* after the detected photon (Sec. S8). This approach allows us to remove the increase in amplitude due to afterpulsing commonly seen in FCS at small correlation times.

## Methods

### Simulations

We simulated both the diffusion of different species of fluorescent molecules through the observation volume and the photon emission process using two different simulators.

#### Continuous space and discrete time

To simulate experiments when the binning time is much less than the diffusion time, we developed a bespoke simulator in Matlab (Mathworks, MA), which uses a discretized time but a continuous representation of space to better describe the observation volume. Diffusion within time bins does not affect the signal statistics because photons from all molecules are generated at the end of a time bin once their positions have been updated. Given an initial concentration, the simulator randomly places molecules in a box of *x* = 4*μ*m by *y* = 4*μ*m by *z* = 8*μ*m with the laser focused at the box’s centre. At each Δ*t*, we sample the emitted photons for each molecule *i* in the observation volume from a Poisson distribution with mean *I*_*i*_(**x**_*i*_). We then independently update the *x*, *y*, and *z* positions of each molecule using Gaussian random variables with variance 2*D*_*i*_Δ*t*. The number of molecules in the volume remains constant: if any of the molecules extend beyond the boundaries of the simulation, they are wrapped to the other side of the simulated volume. We consider two detection channels, with each species potentially having a different brightness in each channel.

#### Discrete space and continuous time

To simulate experiments when the binning time approaches the diffusion time, we modified the reaction-diffusion simulator MesoRD [39]. Molecules can diffuse and fluoresce at any time, and so effects such as diffusion within a bin time, saturation of the excited state, triplet states, detector dead-time, and detector after-pulses can be explored. The simulator uses a discrete space, but continuous time representation. We therefore discretize the intensity profile, and the extent of this discretization affects accuracy.

We split the simulation volume into two compartments: an inner compartment containing the observation volume and an outer compartment for which the laser intensity is low. Each compartment is further divided into thousands of subvolumes (cubes of 25-33 nm per side). Each subvolume has a laser intensity dependent on its position relative to the center of the simulation volume. Following their concentrations, molecules are randomly distributed among the subvolumes. Each species undergoes a set of molecular reactions, such as excitation to the singlet state and relaxation to the ground state. Some reactions result in a photon being emitted and measured in a specified detection channel if a random number generator produces a number smaller than the channel’s detection efficiency. In the outer compartment, molecules undergo a birth-death process, and the number of molecules in the simulation volume fluctuates. Typically, data was recorded in arrival-time mode, with 50 ns resolution and with a simulation time of 60 s. Several simulation runs (usually 5) are used.

We tested the quality of the simulations by fitting the data, similar to a calibration experiment, to ensure that we obtained the correct concentrations, brightness values, diffusion times, structure factor (*s* = *z*_*A*_/*r*_*A*_), and shape parameters (*γ*_3_, *γ*_4_).

#### Images

We extended our simulator to simulate photon detection using a raster-scanned laser scanning microscope. For a *Xμ*m by *Yμ*m image, the simulation volume is a region of *x* = 4*Xμ*m by *y* = 4*Yμ*m by *z* = 8*μ*m. Molecules are placed randomly, and the laser is initially positioned at the top left corner: the region (−*X*/2: *X*/2, −*Y*/2: *Y*/2, 0) of the simulation volume. Each image is assigned *x*_*n*_ by *y*_*n*_ pixels. All pixels have a defined dwell time. At each dwell time, we count photons from all molecules in the simulation volume with the point-spread function centred at the current pixel. These photon counts are entered into the corresponding pixel of the current image frame. The position of each molecule is then updated by diffusion, and the laser moves to the next pixel. At the end of each line, the laser shifts to the next line, but the molecules first have their positions updated over the line retracing time (although in multiple steps with each step having the duration of the laser dwell time). Similarly, the molecules’ positions are updated over the frame retracing time at the end of each frame. The updates are also performed in multiple steps to allow boundary conditions to be considered at each step (molecules that have moved outside the simulation volume are wrapped to the other side).

### Calculating the experimental cPCH

To extract parameters, we must fit the experimental data.

Formatting data for cPCH is straightforward if the data is binned using a single time bin, *T*. For each *τ*, we construct a two-dimensional histogram with photon counts forming one axis of the histogram and photon counts observed a time *τ* later forming the other. Systematically considering all time bins separated by *τ* then allows us to fill the histogram’s bins. The empirical histogram of photon counts for cPCH, denoted $p_{N}^{*}\left(n_{a},n_{b}|\tau ,T\right)$, can then be converted to the empirical probability distribution, $p_{N_{em}}\left(n_{a},n_{b}|\tau ,T\right)$ by normalizing so that its sum is unity. This method can, however, become memory intensive at the smaller bin times of around 200 ns used in FCS.

#### Using arrival-time data

We use arrival-time data to apply cPCH for all the time ranges employed in FCS. It is simplest to work with bin times that are integer multiples of the detector’s sampling time.

Given a list of photon arrival times **t**, we first convert the arrival times and the desired bin time, *T*_*k*_, into integer multiples of the sampling time, i.e. to **t**^*s*^ and $T_{k}^{s}$. We then divide the integer arrival times by the integer bin time, rounding up to the closest integer, to give a list **b**, which indicates in which bin time photons arrive. For example, if **t**^*s*^ = {1, 2, 3, 6, 11, 17, 24, 46} and $T_{k}^{s}=10$, then **b** = {1, 1, 1, 1, 2, 2, 3, 5}, indicating that there were 4 photons in the first bin time, 2 in the second, 1 in the third, 0 in the fourth, and 1 in the fifth. Numerically, this formulation allows the accumulation property of sparse matrices to be used to determine the total photons detected per bin. For the above example, we have that the bins with positive counts are **b**^+^ = {1, 2, 3, 5} with corresponding counts of total photons of {4, 2, 1, 1}. To find the bins at time *τ*, we normalize *τ* by the bin time *T*_*k*_ to obtain *τ*_*k*_. We define $b_{\tau }^{+}$ as $b_{\tau ,i}^{+}=b_{i+\tau_{k}}^{+}$.

To determine $p_{N}^{*}\left(n_{a},n_{b}|\tau ,T\right)$, we first find $p_{N}^{*}\left(n_{a}>0,n_{b}>0|\tau ,T\right)$ by constructing a histogram for all pairs of bins with non-zero photon counts from the intersection of **b**^+^ and $b_{\tau }^{+}$, with the first element of the pair, *n*_*a*_, from **b**^+^ and the second element, *n*_*b*_, from $b_{\tau }^{+}$. Second, when *n*_*a*_ = 0 and *n*_*b*_ > 0 and when *n*_*a*_ > 0 and *n*_*b*_ = 0, we construct a histogram of the photon counts for bins where **b**^+^ and $b_{\tau }^{+}$ do not intersect: for example bins that are in **b**^+^ but not in $b_{\tau }^{+}$ have *n*_*a*_ > 0 and *n*_*b*_ = 0. Finally, for *n*_*a*_ = *n*_*b*_ = 0, we count the number of bin pairs with zero photon counts, which is the difference between the total number of bin pairs and the number of bins with a positive photon count in at least one channel. The empirical probability is calculated by normalizing $p_{N}^{*}\left(n_{x},n_{y}|\tau ,T\right)$ by the total number of bin pairs.

### Calculating the fit energy

We calculate the error of a particular model for a given set of parameters similarly to PCH [8] and model each pair of photon counts {*n*_*x*_, *n*_*y*_} as a binomial experiment with a chance of success of *p*_*N*_(*n*_*x*_, *n*_*y*_|*τ*) and a chance of failure of 1 − *p*_*N*_(*n*_*x*_, *n*_*y*_|*τ*).

#### Fitting the cPCH distribution

If *N*_*d*_ is the number of data points at a particular *τ* and a particular *n*_*x*_ and *n*_*y*_, the standard error, *σ*_*p*_, for the estimate of *p*(*n*_*x*_, *n*_*y*_, *τ*) obeys

$$\begin{array}{c} \sigma_{p}^{2}\left(n_{x},n_{y},\tau \right)=\frac{1}{N_{d}}\times p_{N_{\text{em}}}\left(n_{x},n_{y}|\tau \right)[1-p_{N_{\text{em}}}\left(n_{x},n_{y}|\tau \right)] \end{array}$$ (56)

where $p_{N_{\text{em}}}\left(n_{x},n_{y}|\tau \right)$ is the empirical probability distribution. We note that Eq 56 ignores any correlations between different segments of the dataset.

Writing $L_{n_{x},n_{y}}\left(\tau \right)$ as the total number of histogram entries observed at a given *τ*, we define the fit energy—or loss function—for the cPCH distribution as

$$\begin{array}{c} E_{p}=\frac{1}{\sum_{\tau }L_{n_{x},n_{y}}\left(\tau \right)}\times \sum_{\tau }\sum_{n_{x},n_{y}}\frac{1}{\sigma_{p}^{2}}{\left[(p_{N}\left(n_{x},n_{y}|\tau \right)-p_{N_{\text{em}}}\left(n_{x},n_{y}|\tau \right))\right]}^{2} \end{array}$$ (57)

where we have summed the normalized square error of each entry of the histograms over all *τ*. If the model fits the data within the empirical variance, we expect the energy value to be close to one.

#### Fitting the factorial cumulants

It is often more efficient to fit the factorial cumulants of the data rather than use Eq 57. First, we find the moments *M*_*m*,*n*_(*τ*) from the empirical cPCH distribution using $M_{m,n}\left(\tau \right)=\sum_{n_{x}}\sum_{n_{y}}n_{x}^{m}n_{y}^{n}p_{N_{\text{em}}}\left(n_{x},n_{y}|\tau \right)$. Second, we convert these moments into the cumulants and then into the factorial cumulants (Sec. S1). Third, we determine the empirical variance of each measured factorial cumulant, $\sigma_{K}^{2}\left(\tau \right)$, using moments of moments (Eq 36). Specific expressions, which need only be calculated once per dataset, are in Sec. S9 and are in principle identical to those for dual color FCA and TIFCA [27].

Writing *L*_*m*,*n*_(*τ*) now as the total number of moments used at each *τ*, the fit energy is

$$\begin{array}{c} E_{K}=\frac{1}{\sum_{\tau }L_{m,n}\left(\tau \right)}\times \sum_{\tau }\sum_{m,n}\frac{1}{\sigma_{K}^{2}}{\left[{\left(K\right)}_{m,n}\left(\tau \right)-{\left(K\right)}_{{m,n}_{\text{em}}}\left(\tau \right)\right]}^{2} \end{array}$$ (58)

which is the sum squared error of each factorial cumulant for each *τ* normalized by the total number of moments used.

### Using analytical solutions for the factorial cumulants to sample parameter space

To resolve two species by brightness alone using factorial cumulants, we need to find {*N*_1_, *ϵ*_1_, *N*_2_, and *ϵ*_2_} that solve, within experimental error:

$$\begin{array}{c} {\left(K\right)}_{n}=\gamma_{n}\left(N_{1}{\epsilon_{1}}^{n}+N_{2}{\epsilon_{2}}^{n}\right) \end{array}$$ (59)

from Eq 43. With no other information about our sample, we need to use all factorial cumulants up to *n* = 4.

Eq 59 can be inverted. We find that

$$\begin{array}{ccc} \epsilon_{1} & = & [-\gamma_{2}\gamma_{3}(\gamma_{1}\gamma_{4}{\left(K\right)}_{2}{\left(K\right)}_{3}-\gamma_{2}\gamma_{3}{\left(K\right)}_{1}{\left(K\right)}_{4})\pm \{{\gamma_{2}}^{2}{\gamma_{3}}^{2}(\gamma_{1}\gamma_{4}{\left(K\right)}_{2}{\left(K\right)}_{3}\\ & & -\gamma_{2}\gamma_{3}{\left(K\right)}_{1}{\left(K\right)}_{4}{)}^{2}+4\gamma_{1}\gamma_{2}\gamma_{3}\gamma_{4}(-\gamma_{1}\gamma_{3}{{\left(K\right)}_{2}}^{2}+{\gamma_{2}}^{2}{\left(K\right)}_{1}{\left(K\right)}_{3})\times \\ & & \left(\gamma_{2}\gamma_{4}{{\left(K\right)}_{3}}^{2}-{\gamma_{3}}^{2}{\left(K\right)}_{2}{\left(K\right)}_{4}){\}}^{\frac{1}{2}}]/[2\gamma_{3}\gamma_{4}(-\gamma_{1}\gamma_{3}{{\left(K\right)}_{2}}^{2}+{\gamma_{2}}^{2}{\left(K\right)}_{1}{\left(K\right)}_{3})\right] \end{array}$$ (60)

with

$$\begin{array}{c} \epsilon_{2}=\frac{\gamma_{1}\left[\epsilon_{1}\gamma_{3}{\left(K\right)}_{2}-\gamma_{2}{\left(K\right)}_{3}\right]}{\gamma_{3}\left[\epsilon_{1}\gamma_{2}{\left(K\right)}_{1}-\gamma_{1}{\left(K\right)}_{2}\right]} \end{array}$$ (61)

and

$$\begin{array}{ccc} N_{1}=-\frac{\epsilon_{2}\gamma_{2}{\left(K\right)}_{1}-\gamma_{1}{\left(K\right)}_{2}}{\epsilon_{1}\left(\epsilon_{1}-\epsilon_{2}\right)\gamma_{1}\gamma_{2}} & ; & N_{2}=\frac{{\left(K\right)}_{1}-\epsilon_{1}\gamma_{1}N_{1}}{\epsilon_{2}\gamma_{1}}. \end{array}$$ (62)

There is a single, unique solution because the two solutions are symmetric. Statistical errors in the empirical factorial cumulants can result in non-physical (negative) values for these analytical solutions.

Nevertheless, the analytical solutions do provide an efficient method to sample brightness and concentration values that are within the experimental error of the data. We use the method of moments of moments to estimate the error (variance) for each factorial cumulant in terms of the empirical raw moments following Eq 36. Once this variance is estimated and assuming the empirical factorial cumulants obey a Gaussian distribution with a mean equal to their empirical value and a variance given by Eq 36, we can resample each moment within the expected experimental error and use Eqs 60 to 62 to estimate the corresponding values of the parameters.

Not only do these samples speed up fitting by providing initial values for any optimization algorithm but they also allow us to visualize both the parameter space and the ‘energy’ landscape that the fitting algorithm explores (Sec. S2 and [35]). This visualization can indicate, for example, that more data is needed to reduce the size of the relevant region of parameter space (by reducing the error on the fourth factorial cumulant) or that cPCH should be used to resolve the two species by their diffusion too.

### The fitting algorithm

In fluorescence fluctuation spectroscopy, the fit energy is often minimized using algorithms for nonlinear least-squares [8, 11, 28]. In our hands, such algorithms (Levenberg-Marquardt) perform poorly for brightness analysis, including cPCH, probably because the parameter space has multiple local minima.

Given the rugged energy landscape, we minimize the fit energy using nested sampling. Nested sampling is a Markov chain Monte Carlo method where multiple sets of parameters are simultaneously optimized, with the worst solutions being replaced at every iteration by randomly perturbing another solution [40, 41].

Briefly (for more details see [35]), we:

1. Sample (typically 100) initial sets of parameters from empirical factorial cumulants using the inverted solutions (Eqs 60–62);
2. Rank each set of parameters by its energy using Eq 58;
3. Record the parameter set with the highest (worst) energy and replace these parameters with a randomly chosen copy of another parameter set;
4. Update the copied parameter set, one parameter at a time, using 20 iterations from an update distribution (either a normal or, for the diffusion coefficient, log normal distribution). If the parameters are still within their bounds, always accept if the new energy is less than *E*_min_ or otherwise accept using a Metropolis step;
5. Either decrease the step size for this parameter set by the exponential of the reciprocal of the number of accepted steps if the number of accepted steps is greater than the number of rejected steps or increase the step size by the exponential of the reciprocal of the number of rejected steps if the number of rejected steps is greater than the number of accepted steps;
6. Remove and record the parameter set with the next highest energy and update this parameter set using the steps above;
7. Terminate if the desired number of iterations has passed, the energy has decreased to a specified value, or the parameters no longer change beyond a certain threshold.

We use the average of each of the recorded parameters to find the best-fit values (after an appropriate burn-in).

We adjusted the acceptance step to have an *E*_min_ (typically set to 2) to explore parameter sets that are compatible with the data within the expected error rather than only lying in the region with the lowest energy. Note that because parameter sets can have symmetric solutions (by swapping the parameter sets for species 1 and 2), we enforced one parameter in one species to be greater than or equal to the same parameter in the other.

## Results

**Fig 1:**
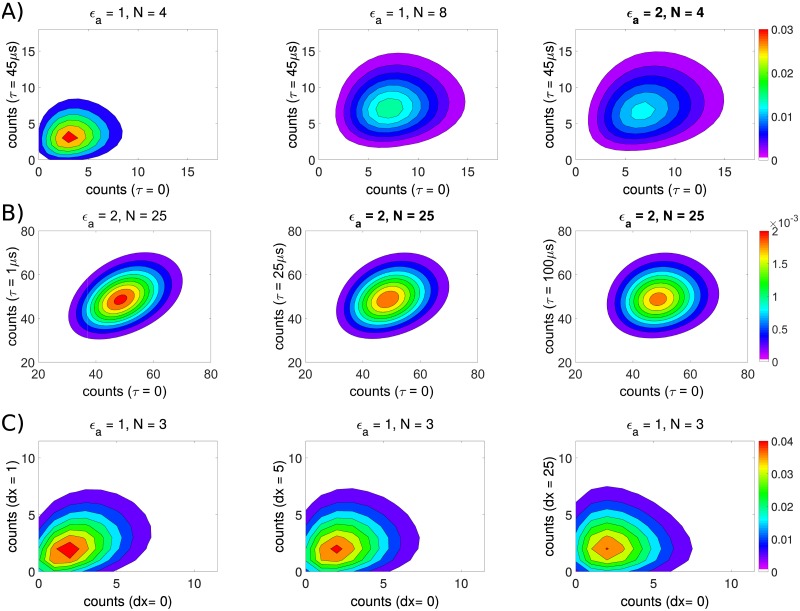
cPCH distributions are sensitive to the concentration, brightness, and diffusion of the fluorophores with a higher probability of photon counts near the *n*_*x*_ = *n*_*y*_ axis when *τ* < *τ*_*d*_. A) cPCH distributions at different concentrations and brightnesses at *τ* = *τ*_*d*_ (the diffusion time, *τ*_*d*_, is 45 *μ*s). B) cPCH distributions at different time shifts *τ* for a single species with a concentration of *N* = 25 molecules per confocal volume and a diffusion time of *τ*_*d*_ = 45 *μ*s. The axial-to-radial beam waist ratio is *s* = 5, and the time-shifted channel is from the same spectral channel as the time 0 channel. C) Spatial cPCH distributions at different pixel separations (1, 5, and 25 horizontal pixels), using a diffusion coefficient of *D* = 100 *μ*m^2^/s, a pixel size of *δ*_*x*_ = *δ*_*y*_ = 40 nm, a pixel dwell time of *τ*_*p*_ = 10 *μ*s, and an image size of 512 × 512 pixels.

The shape of the cPCH distribution changes with the concentration, brightness, and diffusivity of the fluorophores and with the time difference, *τ*, between the two detection channels (Fig 1). Let *τ*_*d*_ be the time taken for a fluorophore to diffuse across the observation volume. Then for small *τ*/*τ*_*d*_, there is a strong correlation between counts in the *τ* = 0 channel and counts in the time shifted channel (Fig 1B). Molecules have not had time to diffuse to a region of different intensity, and correspondingly there is a higher probability of identical photon counts in each channel. As the *τ*/*τ*_*d*_ ratio increases, the correlation between *n*_*x*_ and *n*_*y*_ counts decreases, and the histogram becomes more symmetric.

### FCS, moment analysis, and PCH are special cases of cPCH

**Fig 2:**
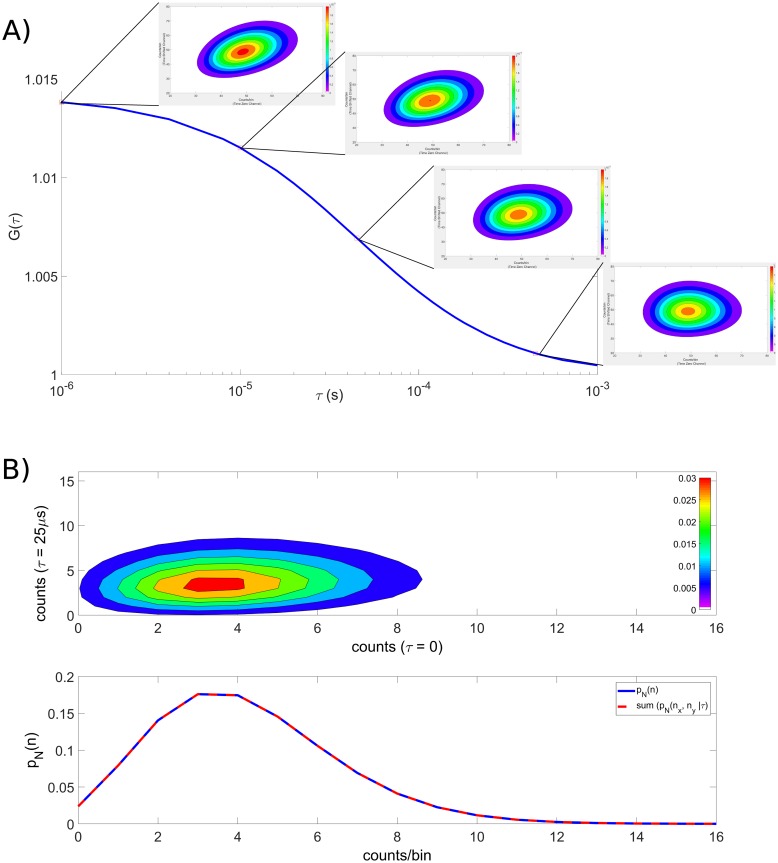
cPCH can recover the results of both FCS and PCH. A) Obtaining an FCS curve from cPCH data: a single point on an FCS curve, *G*_FCS_(*τ*), is calculated from the normalized moments of the cPCH distribution, *p*_*N*_(*n*_*x*_, *n*_*y*_|*τ*) (parameters from Fig 1B). B) Obtaining a PCH distribution: summing over one of the channels of the cPCH distribution gives the PCH (parameters are *ϵ*_*a*_ = 0.7, *N* = 6, *τ*_*d*_ = 50 *μ*s, and *s* = 5, with a time shift of *τ* = 25 *μ*s).

From the same data, cPCH can recover the results of FCS [7], moment analysis [11], and PCH [8, 9]. Similarly, scanning FCS [42, 43] and RICS [24, 36] can be considered special cases of spatial cPCH. We generate a single point on an FCS curve from the joint first moment of the cPCH distribution, *M*_1,1_(*τ*), normalized by the mean of each channel (Eq 39 and Fig 2a), and the PCH distribution by summing over all the counts in either channel of the cPCH distribution (Eq 42 and Fig 2b). We can determine the factorial cumulants obtained using FCA by setting either *m* or *n* to zero in the factorial cumulants (*K*)_*mn*_(*τ*) (Eq 33). Finally, the dual color PCH is equivalent to the dual color cPCH distribution at *τ* = 0 (Eq 44).

### Spatial cPCH

Extending FFS techniques to images enables the analysis of membrane-bound and immobile proteins, and we compare the photon counts from one pixel in the image to those of another in spatial cPCH. For raster-scanned images, these pixels are temporally separated by the time the laser needs to move between the two. The probability of detecting a correlation between photons from the first pixel and photons from the second pixel depends on the fluorescent molecule being able to move from the first pixel to the second in this time. The probability therefore depends on the distance between the two pixels, the size of the pixels, the molecule’s diffusion, and the scan rate of the laser (Eq 54).

As in cPCH, the spatial cPCH distributions show higher probabilities along the *n*_*x*_ = *n*_*y*_ axis at short correlation times (corresponding to short distances between pixels) because there is a high probability that the molecules will occupy a region of similar intensity in the later pixel (Fig 1C). As the distance between pixels increases, the molecules are no longer likely to be in regions of similar intensity, and the distribution becomes more symmetric (for the single color case). Like scanning FCS [44] and RICS [24, 36], spatial cPCH can be used to directly estimate the diffusion coefficient, rather than the diffusion time. Using spatial cPCH, we can also measure the brightness of immobile molecules (S3 Fig) and so distinguish between the image’s mobile and immobile fractions.

### Validation using a single species

To validate cPCH, we simulated a single fluorescent species diffusing through a Gaussian observation volume. To ensure that interpreting the analysis is unaffected by sampling errors, we simulated a long (14 minute) experiment, but we emphasize that in practice cPCH requires similar amounts of data as other FFS methods. We fit both the cPCH and the factorial cumulants to the simulated data. Similar to a calibration measurement, we also estimate parameters that characterize the observation volume (the shape factors *γ*_3_ and *γ*_4_ from Eq 11 with *γ*_*m*+*n*_ = *γ*_*m*,*n*_ and the structure factor *s* = *z*_*A*_/*r*_*A*_ from Eq 7).

**Fig 3:**
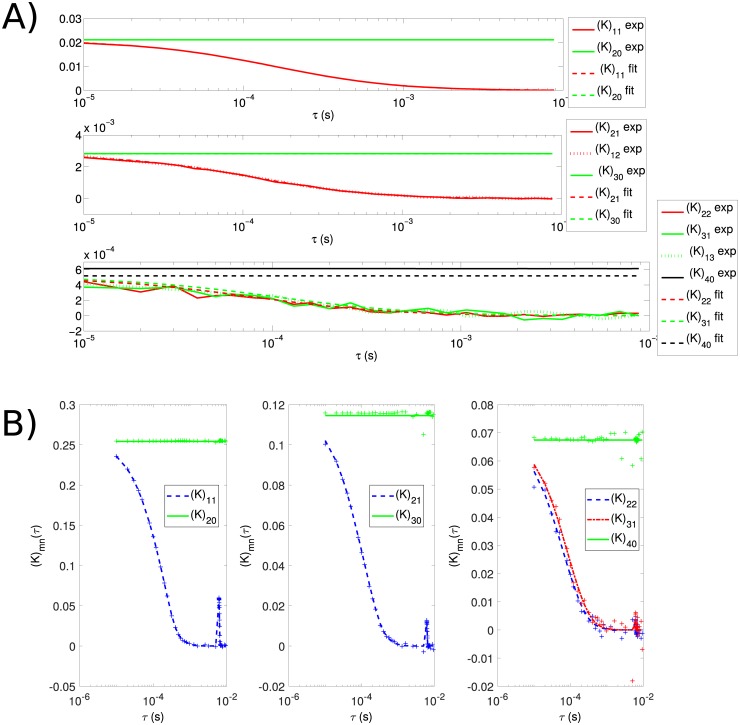
cPCH allows accurate inference of the factorial cumulants for single fluorescent species and mixtures of species with different rates of diffusion. A) Fitting the factorial cumulants of the cPCH distribution gives strong agreement between the empirical factorial cumulants estimated from simulated data and those calculated with the best-fit parameters (using Eq 33 and the parameters in Table 1). For the lower moments, the empirical and estimated factorial cumulants overlap. B) Fitting the factorial cumulants of the spatial cPCH distribution gives similar strong agreement (crosses denote the empirical factorial cumulants and the dashed lines show the fits). We consider a mixture of a slowly (*D* = 0.1 *μ*m^2^/s) and a quickly (*D* = 200 *μ*m^2^/s) diffusing species.

Inference of both the factorial cumulants and the biophysical parameters is accurate (Table 1). The minimum values of the fit energies are close to 1, which is similar to their values evaluated with the true parameters. We therefore conclude that the approximations behind cPCH adequately describe the behavior of the simulated fluorescent molecules. Correspondingly, the empirical factorial cumulants match those calculated from Eq 33 using the inferred values of the parameters (Fig 3A). As expected, the higher order factorial cumulants have more statistical uncertainty.

**Table 1:** cPCH allows accurate inference of biophysical parameters from simulated data. Simulations are from Fig 3A with a bin time of *T* = 10 *μ*s, and the best-fit values found using the factorial cumulants.

**Parameter**	**Actual value**	**Estimated value (estimated/actual)**
*N*	1	1.001 ± 0.002 (1.001)
*ϵ*	0.24448 counts/bin	0.2442 ± 0.0004 counts/bin (0.998)
*γ*_3_	0.192	0.194 ± 0.002 (1.008)
*γ*_4_	0.125	0.146 ± 0.003 (1.167)
*τ*_*d*_	175.7 *μ*s	178 ± 2 *μ*s (1.01)
*s*	1.928	1.82 ± 0.04 (0.94)
**Energy type**	**Energy using actual parameters**	**Energy using fit parameters**
*E*_*K*_	0.993	0.649
*E*_*p*_	0.920	0.823

We note that we do not normalize the factorial cumulants (unlike in higher order correlation analyses [16]) because we find that the un-normalized factorial cumulants have better sensitivity to brightness, allow more cumulants to be fit, and permit the use of analytical solutions to speed-up the fitting algorithm (Methods).

To test spatial cPCH, we simulated molecules diffusing in a three dimensional box whose centre was scanned with a simulated laser-scanning microscope. Tests using a single species achieved similar accuracy to the results for regular cPCH (Table 2). The secondary peaks in the factorial cumulants occur when slowly moving molecules are sampled again on subsequent lines of the image and can be seen in both the theoretical and simulation results (Fig 3B).

**Table 2:** Spatial cPCH can achieve similar accuracy as cPCH. The simulation has a duration of 25 frames and uses an image size of 256 × 256 with a pixel size of *δ*_*x*_ = *δ*_*y*_ = 11.7 nm, a pixel dwell time *τ*_*p*_ of 10 *μ*s, a line retracing time *τ*_*L*_ of 1 ms, and a frame retracing time of 5 ms.

Parameter	Actual Value	Fit Value (fit/actual)
*N*	0.6	0.599 (1.002)
*ϵ*	1.1 counts/bin	1.102 counts/bin (0.999)
*γ*_3_	0.192	0.196 (1.018)
*γ*_4_	0.125	0.128 (1.025)
*r*_*o*_	265.1 nm	267.6 nm (1.010)
*D*	100 *μ*m^2^/s	100.5 *μ*m^2^/s (0.995)
*s*	1.93	1.78 (0.93)

### Resolving different species using cPCH

**Fig 4:**
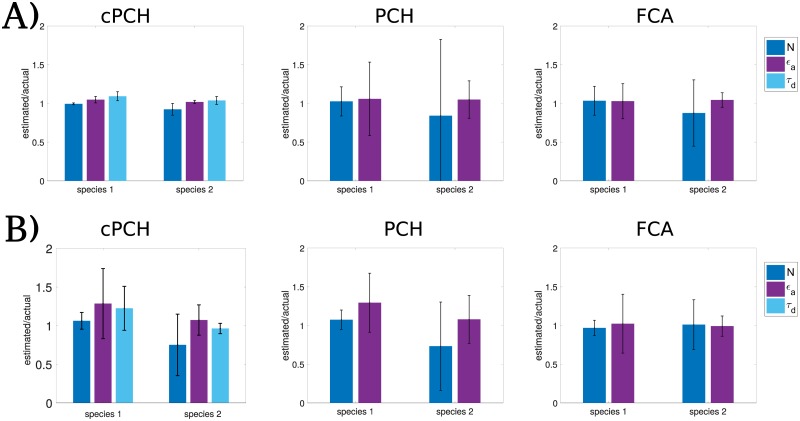
If both brightness and diffusion can be used to resolve the two species, cPCH can both resolve different species better than PCH and FCA and determine diffusion times. A) Inference from 60 s of simulated data of two different species showing the ratio of the estimated parameters to the actual parameters when both brightness and diffusion differ between the two species. Parameters are: *N*_1_ = 8, *ϵ*_1_ = 48895 counts/s, and *τ*_1_ = 87.85 *μ*s for species 1; *N*_2_ = 2, *ϵ*_2_ = 146686 counts/s, and *τ*_2_ = 439.24 *μ*s for species 2. The bin time is *T* = 10 *μ*s. B) Results of inference from 75 s of simulated data when only brightness substantially differs between the two species. Parameters are: *N*_1_ = 8, *ϵ*_1_ = 48895 counts/s, and *τ*_1_ = 21.96 *μ*s for species 1; *N*_2_ = 5, *ϵ*_2_ = 146686 counts/s, and *τ*_2_ = 25.16 *μ*s for species 2. The bin time is *T* = 10 *μ*s.

cPCH is better able to resolve two fluorescent species than both PCH and FCA because cPCH uses information on both diffusion and brightness. To determine the cPCH’s ability to resolve, we simulated a mixture of two different species with the brightness of one species three times that of the other. When the two species have different diffusion coefficients and different brightnesses, not only does cPCH outperform PCH and FCS (Fig 4A) but also allows more accurate inference of all parameters. We see a similar improvement in accuracy for a mixture of fluorophores with one species twice as bright as the other (S5 Fig). When the two species have close diffusion coefficients but different brightnesses, cPCH performs as well as PCH and FCA (Fig 4B), although also allowing inference of the diffusion times.

cPCH performs at least as well as PCH and FCA in other tests too. We simulated a mixture of two different species with the brightness of one species double that of the other. In this case, both fluorophores are relatively dim, but the brighter fluorophore outnumbers the dimmer fluorophore, a scenario that is challenging for FFS methods (S6 Fig). With cPCH, we could resolve the concentration of the dimmer species, but with less precision than before. Nevertheless, cPCH outperforms PCH, FCA, and FCS (S6 Fig). We also determined the ability of spatial cPCH to resolve two species (Fig 3B and S4 Fig), with similar performance.

### Binning effects

It is sometimes necessary to use larger binning times to improve the signal to noise ratio when the molecular brightness is low. By using larger binning times, however, the individual molecules have time to diffuse to locations of different intensity within a bin time, and so diffusion effects must be included in the analysis.

**Fig 5:**
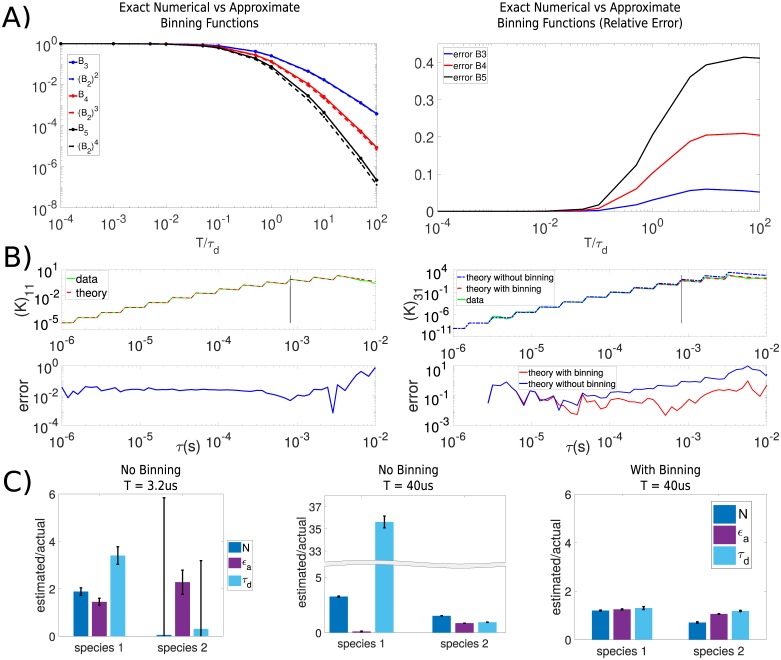
cPCH also performs well for large binning times if approximations to the binning functions of TIFCA are used. A) Approximations of the binning functions (the TIFCA approximation used in FIMDA—Eq 49) are accurate up to third order. We employ bin sizes that increase every 4 *τ* values and range from *T* = 200 ns to *T* = 800 *μ*s. B) Binning functions improve estimates of factorial cumulants. Factorial cumulants estimated empirically (solid lines) from a single species simulated with a diffusion time of *τ*_*d*_ = 175.7 *μs* are compared with the corresponding analytical results (dashed lines from Eq 33). The black vertical line indicates the *τ*(*s*) at which the binning time exceeds the diffusion time across the observation volume. Binning functions are used to improve the estimate of (*K*)_31_ but are not required for (*K*)_11_. C) The binning approximations improve inference of biophysical parameters. We simulated the diffusion of two low brightness species for 3 minutes, each at 5 nM with *ϵ*_1_ = 17780 counts/s and *ϵ*_2_ = 35560 counts/s and with the diffusion time of the second species four times slower than the first (43.92 *μ*s), and then used cPCH to infer parameters. For a bin time of *T* = 3.2 *μ*s (much less than the shortest diffusion time of the two species) giving only 0.057 and 0.114 counts/bin, inference is correspondingly poor. For *T* = 40 *μ*s giving 0.711 and 1.422 counts/bin, inference is only accurate if we use the binning approximation.

In cPCH, we apply the FIMDA binning approximation (Eq 48) to higher order binning functions (Eqs 49 and 50). This approximation works well up to the third order binning function (Fig 5A). The greater statistical uncertainty in the measurements of the higher order moments (we consider up to fourth order) mean that these moments receive less weight when fitting the factorial cumulants in cPCH, and the errors caused by the approximation have little impact.

In simulations, including the binning functions improves both empirical estimates of the factorial moments (Fig 5B) and inference of biophysical parameters (Fig 5C), even when *T* < *τ*_*d*_.

### Resolving mixtures of species with multiple binding sites

Finally, we demonstrate that cPCH can work in a scenario where FCS, PCH, and FCA are all unable to resolve the different species. Consider a receptor with two different binding sites, each with its own affinity for a fluorescent ligand, and with the receptor diffusing slower than the ligand. There are three different fluorescent species—unbound ligand (denoted *L*), receptors with a single bound ligand (*R*_1_), and receptors with two bound ligands (*R*_11_)—and one non-fluorescent species—unbound receptors (*R*). FCS cannot differentiate between the singly- and doubly-bound receptors (*R*_1_ and *R*_11_) because their diffusion coefficients do not differ (being determined by the more massive receptor); PCH and FCA cannot differentiate between the unbound ligand and the singly-bound receptors (*L* and *R*_1_) because both have the same fluorescence.

We simulated this system at equilibrium using an initial amount of ligand *L*_*o*_ and analyzed the data using spatial cPCH. From the empirical histograms of spatial cPCH, we calculated and then fit the factorial cumulants using a model with three fluorescent species (*L*, *R*_1_, and *R*_11_; Sec. S5). The concentration of *R* can be estimated if *R*_0_, the initial concentration of free receptor, is known, via *R*_0_ = *R* + *R*_1_ + *R*_11_.

**Fig 6:**
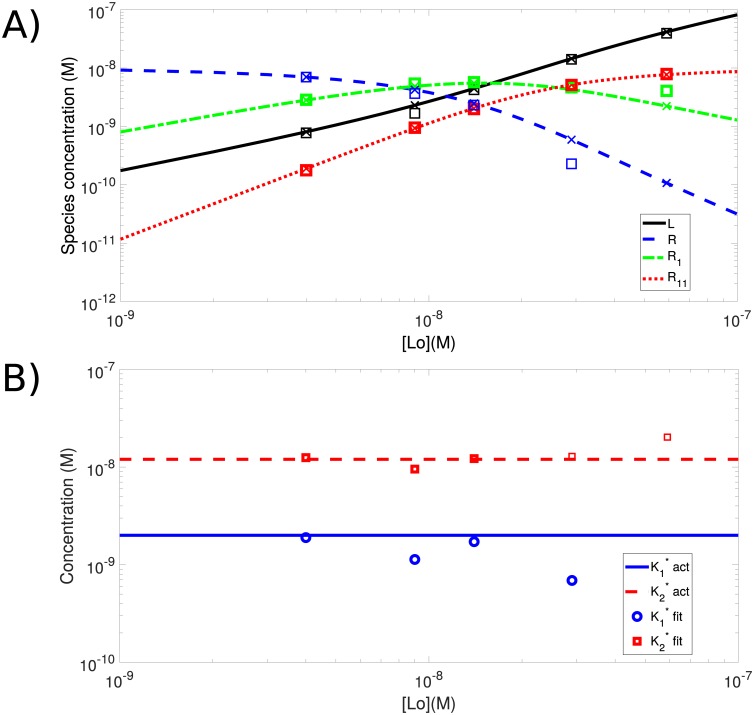
Spatial cPCH can be used to estimate the dissociation constants in a receptor-ligand system with two different binding sites on the receptor and a fluorescent ligand. A) Spatial cPCH can accurately estimate concentrations if the concentration of the doubly-labeled receptor *R*_11_ is lower than that of the singly-bound receptor *R*_1_. The actual concentrations are indicated with crosses and the estimated concentrations are indicated by the open squares. The initial concentration of receptors is *R*_0_ = 10 nM. We specified the (apparent) dissociation constants for the singly and doubly bound receptors as $K_{1}^{*}=2\;\text{nM}$ and $K_{2}^{*}=12\;\text{nM}$ and find the actual dissociation constants from Eq S9. These apparent dissociation constants arise because we cannot distinguish using either fluorescence or diffusion which site the ligand has bound on a receptor that has bound only one ligand (Sec. S5). The simulation employed a laser dwell time of 10 *μ*s with images of 512 × 512 pixels and an image size of 4 *μ*m by 4 *μ*m, so that the pixel size is just under 12 nm. The line retracing time is 1 ms and the frame retracing time is 50 ms. We generated 2 sets of data with 25 images each for all of the concentrations used, except for the lowest initial value of ligand, which used 1 set of 70 images. B) The apparent dissociation constants ($K_{1}^{*}=LR/R_{1}=2\;\text{nM}$ and $K_{2}^{*}=LR/R_{11}=12\;\text{nM}$) can be estimated using the measured concentrations provided the concentration of singly labeled receptors *R*_1_ is greater than the concentration of doubly labeled receptors *R*_11_.

Spatial cPCH is able to accurately estimate all four concentrations, provided we use concentrations of ligand below the concentration at which *R*_1_, the singly bound receptors, reaches its maximum (Fig 6A). At higher concentrations of ligand, the doubly-bound receptor, *R*_11_, and free ligand, *L*, dominate the signal, and *R*_1_ is difficult to estimate. Errors in *R*_1_ propagate through to errors in the estimates of *R*, and both errors affect estimates of the dissociation constant $K_{1}^{*}=\frac{LR}{R_{1}}$ (Fig 6B). An alternative is to directly estimating *R* using a dual-color assay. We note that we could also use cPCH (rather than spatial cPCH) because both receptors and ligand are freely diffusing. If receptors are confined to a membrane, spatial cPCH is the preferred technique.

If either FCS or PCH are used instead of cPCH, estimates of the different concentrations would have to be deduced from the overall behaviour of the fluctuations in fluorescence as a function of concentration, making the task more difficult [45].

## Discussion

We have introduced cPCH, which unifies several FFS techniques (FCS, PCH, and FCA) to resolve different molecular species through both their brightness and diffusion. PCH and dual-color PCH are special cases of the cPCH distribution; FCS and FCA are special cases of the moments of the cPCH distribution.

Although data can be fit to the cPCH distribution, we usually find fitting its factorial cumulants to be more computationally efficient. Using simulated data, we have shown that the factorial cumulants can resolve multiple species at higher concentrations than analyses using the distributions [35]. Nevertheless, there are situations where the distribution can be useful, such as correcting for dead-time and afterpulsing (Sec. S7, S8 and [35]) and determining the shape parameters *γ*_1_ and *γ*_2_ [31]. Further, it is straightforward to sample from the discrete probability distributions providing a convenient method to generate empirical distributions of the photon counts at a given *τ* and *T* for testing statistical properties of the cPCH distributions, numerical implementations, etc.

In addition, we have developed a novel fitting algorithm based on a Markov chain Monte Carlo scheme for nested sampling. To improve the algorithm’s efficiency, we select initial parameters by inverting and sampling from the analytical solutions to the factorial cumulants (using theoretical expressions for the variance of each moment for the sampling). With this method, we can visualize the parameter space and initialize optimization algorithms in the relevant regions of parameter space.

We show that cPCH resolves two differently diffusing species. cPCH reduces the uncertainty in fitting if the two species have different diffusion and brightness and performs as well as both PCH and moment analysis when the two species differ only in their brightness. For example, resolving two different species by brightness alone is challenging because fourth order moments are required, which are difficult to estimate. cPCH circumvents this problem by also being sensitive to differences in diffusion for which only second-order moments are needed. As PCH and FCS are special cases of cPCH, we can also always choose to use one of these techniques instead of cPCH without having to reformat the data.

Our work is related to that of Melnykov et al. [16]. Their expressions for the higher-order factorial cumulants, although derived differently, correspond to the factorial cumulants of the cPCH distribution. Nevertheless, they normalize these bivariate expressions by the univariate factorial cumulants to allow better comparison with FCS. Our un-normalized factorial cumulants allow us both to more efficiently explore parameter space by exploiting the analytical solutions for the univariate factorial cumulants during fitting and to constrain the diffusion time if we apply a series of bin sizes up to those large relative to the diffusion time (following FIMDA [46] and TIFCA [18]).

Abdollah-Nia et al. have recently employed normalized higher-order FCS to study reaction kinetics [47, 48]. They show that by normalizing the higher order factorial cumulants of the reacting system with those of a non-reacting system, the effects of diffusion and the illumination profile can be removed, isolating the reaction kinetics. This method is potentially powerful for studying reaction kinetics, but for species with similar diffusion. We have focused on resolving different species based on differences in their brightness and diffusion and have demonstrated cPCH with a ligand-binding system with two distinct binding sites, where the ligand and receptor diffuse differently. Such a system would be unamenable to the analysis of Abdollah-Nia et al.

Another recent development is 2D fluorescence lifetime correlation spectroscopy (2DFLCS), which differentiates molecules based on their diffusion and fluorescence lifetimes and can analyse molecular conformations on the microsecond scale [49–51]. Although similar to cPCH because 2DFLCS uses histograms of the fluorescence lifetimes of photons detected at a time *τ* apart, it, unlike cPCH, requires a setup for time-correlated single-photon counting. Indeed, cPCH is able to differentiate molecules based on their brightness even if their fluorescence lifetimes are identical, as expected, for example, for fluorescent oligomers. As Fluorescence Intensity and Lifetime Distribution Analysis (FILDA) combines fluorescence lifetime analysis with photon counting histograms [52], we expect that cPCH could be combined with 2DFLCS to allow molecules to be differentiated by fluorescence lifetime, brightness, and diffusion.

With a simple modification, cPCH can be extended to the study of images. Spatial cPCH, like RICS [24] and scanning FCS [44], but unlike FCS and cPCH, is able to directly estimate the diffusion coefficient of the fluorophores (rather than their diffusion time, which depends on both the diffusion coefficient and the beam waist parameter *r*_*A*_). Knowing diffusion coefficients is useful for calibrating optical systems and for studying fluorescent dyes and proteins.

Although we have focused on raster scanning, the derivation of spatial cPCH would apply too to scanning FCS and directed flow. By scanning the laser over a range of positions, spatial cPCH is also able to sample more molecules in a shorter period of time. Spatial cPCH can therefore be used to quantify the concentrations of immobile fluorophores—a challenge for FCS and cPCH because such fluorophores quickly photobleach with a stationary illumination profile and only produce photon counts that are Poisson-distributed. Future work could include the thickness profile of cells by using *z*-scanning [53].

Both cPCH and spatial cPCH require photon-counting detectors. Spatial intensity distribution analysis (SpIDA) [25] is an extension of brightness analysis to images, but employs a calibration method to allow the use of analog photomultiplier tubes. Spatial cPCH can also be extended to analog detectors [35].

We have used simulations to verify cPCH and spatial cPCH, but, in practice, detectors often introduce artefacts because of dead-time and afterpulsing, and experiments can be undermined by triplet states and blinking of fluorophores. The theory of cPCH and spatial cPCH can include such effects (Secs. S6-8). Although we assume a 3D Gaussian observation volume (a similar assumption was made in TIFCA [18] and higher-order FCS [16] and adequately describes diffusion but not the shape parameters of the observation volume), our derivation is general, and it is possible to use the shape parameters of a Gaussian-Lorentzian observation volume for *γ*_*m*,*n*_ and use numerical correlation functions for *G*_*m*,*n*_(*τ*, *τ*_*d*_). In our simulated calibration, we optimized the shape parameters *γ*_3_ and *γ*_4_.

cPCH is valid for both single-channel and dual-color analysis. The combined use of two colour channels and information on diffusion can substantially increase accuracy in resolving multiple species by further reducing the requirement for the third and fourth order moments and by causing the joint factorial cumulants to be determined by only interacting species (labeled with a fluorophore from each channel). We therefore expect dual-color cPCH to be the natural choice for studies that consider interactions between labelled molecular species.

In summary, we anticipate that cPCH and spatial cPCH will become powerful techniques for quantitative, single-cell biology.

## Supporting information

S1 Fig There are many solutions that have energies low enough to be considered within the experimental uncertainty of the data. Scatter plots of the energy of physically valid solutions sampled from inverting the factorial cumulants for a simulation of a mixture of monomers and dimers with *N*_1_ = 3, *ϵ*_1_ = 0.4, *N*_2_ = 2, and *ϵ*_2_ = 2*ϵ*_1_. Each blue dot represents the energy corresponding to a sampled solution; the red asterisk shows the energy corresponding to the actual parameters.
(EPS)

S2 Fig By measuring ϵ_1_ *a priori*, there is a minimum in the energy as a function of the parameter values. Scatter plots of the energy for physically valid samples for the same data of S1 Fig.
(EPS)

S3 Fig As immobile and mobile fluorophores show different decays in their joint factorial cumulants, spatial cPCH can distinguish one from the other. Plots of the higher order spatial factorial cumulants for a mobile (A) and an immobile species (B). Only the diffusion coefficients have different values.
(EPS)

S4 Fig Spatial cPCH can resolve two species with different diffusion coefficients. Inference using a spatial cPCH fit of the factorial cumulants of simulated imaging data using two sets of 25 frames each and with a quickly and a slowly diffusing species (*ϵ*_1_ = 31181 counts/s, *N*_1_ = 1.2, *D*_1_ = 0.1 *μ*m^2^/s, *ϵ*_2_ = 95452 counts/s, *N*_2_ = 0.6, and *D*_2_ = 200 *μ*m^2^/s). The fitting algorithm only considered *τ* up to 0.01 s and so was unable to resolve *D*_1_ because *τ*_1_≈ 0.176 s.
(EPS)

S5 Fig cPCH can outperform alternative methods: A mixture of fluorophores, with one species twice as bright as the other. We simulated data for 240 s and show the ratios of the fit results to the true parameters (*N*_1_ = 8, *ϵ*_1_ = 48895 counts/s, *τ*_1_ = 175.7 *μ*s, *N*_2_ = 2, *ϵ*_2_ = 2*ϵ*_1_, and *τ*_2_ = 351.39 *μ*s and a bin time of 10 *μ*s).
(EPS)

S6 Fig cPCH can out perform alternative methods: Two dim species. Analysis of data for a mixture of two dim species, with the brighter species outnumbering the dimmer species, simulated for 210 s. The ratio of the fit results to the actual parameters are shown (*N*_1_ = 0.4, *ϵ*_1_ = 14669 counts/s, *τ*_1_ = 175.7 *μ*s, *N*_2_ = 0.9, *ϵ*_2_ = 39116 counts/s, *τ*_2_ = 43.9 *μ*s and a bin time of *T* = 10 *μ*s).
(EPS)

S7 Fig Dead-time corrections are necessary when dead-time is present. A nested sampling fit to a MesoRD simulation employing a 50 ns dead-time: **A)** without or **B)** with dead-time corrections. For this simulation, *N*_1_ = 0.213, *ϵ*_1_ = 88900 counts/s, and *τ*_1_ = 43.92 *μ*s for species 1, and *N*_2_ = 0.213, *ϵ*_2_ = 177800 counts/s, and *τ*_2_ = 175.7 *μ*s for species 2. The maximum bin size is *T* = 3.2 *μ*s and the detector dead-time was 50 ns.
(EPS)

S8 Fig Correcting for afterpulses. Simulated calibration data used to estimate *p*(*τ*) and a demonstration of the removal of afterpulsing effects between time bins for an afterpulse probability q* = 0.01 and an average arrival time *τ*_ap_ = 200 ns. **A)** Histograms of arrival times and afterpulses. **B)** Fit to the measured *p*(*τ*). **C)** A comparison of the calculated FCS curves for a 1 nM solution with a diffusion coefficient of 1 × 10^−10^ m^2/^s, with afterpulsing, without afterpulsing, and with afterpulsing removed (using simulated data). The black dotted lines show the FCS curves of the original data without afterpulsing; the green curves show the FCS curves with afterpulsing added to the data; the dashed red curves show the afterpulse-corrected FCS curve using the measured *p*(*τ*); and the blue curves show the afterpulse-corrected FCS curve using the ideal *p*(*τ*).
(EPS)

S1 Table Summary of the simulated calibration results including dead-time effects, triplet states, and binning effects. Both triplet and diffusive binning are necessary. In the third column, the minimum bin size was *T* = 200 ns and the maximum was *T* = 3.2 *μ*s. In the fourth column, the minimum bin size was *T* = 1 *μ*s and the maximum was *T* = 64 *μ*s. * Both diffusion and triplet binning included in the fit. ** Only diffusion binning included in the fit. *R* denotes the triplet fraction; *τ*_*t*_ denotes the triplet relaxation time.
(PDF)
