# Correction: Resolving fluorescent species by their brightness and diffusion using correlated photon-counting histograms

**DOI:** 10.1371/journal.pone.0320511

**Published:** 2025-03-18

**Authors:** Nathan Scales, Peter S. Swain

There are errors in the funding statement. The correct funding statement is as follows: The authors gratefully acknowledge the financial support from the Natural Sciences and Engineering Research Council of Canada (NSERC) (Recipients: NS and PSS) and the Biotechnology and Biological Sciences Research Council (BBSRC) (Grant No. BB/M024881/1, awarded to PSS).

In the Moments of moments subsection of Theoretical foundations, there is an error in the 36^th^ equation. A backslash character is missing in the latex code for one of the summations. Please view the complete, correct equation here:


$$\text{Var}\left[X_{p,r}\right]≃\overset{p}{\underset{x,u=0}{\sum }}\overset{r}{\underset{y,v=0}{\sum }}\frac{\partial X_{p,r}}{\partial M_{x,y}}\frac{\partial X_{p,r}}{\partial M_{u,v}}\text{Cov}\left[M_{x,y},M_{u,v}\right]$$

S1 File is omitted from the list of Supporting Information. This file provides important details on the practical implementation of correlated photon-counting histograms (cPCH): it shows how to convert experimental raw moments into factorial cumulants and vice versa, using Stirling numbers and recursion relations, in order to relate empirical data to theoretical predictions; it provides additional theoretical background for the receptor-ligand binding system with multiple binding sites; it provides corrections for cPCH for dark and triplet states, as well as for detector dead-times and afterpulsing; finally, it provides expressions for the variance of the factorial cumulants, calculated using the moments of moments method and the recursion relations between moments. The Figs S1–S8 are also reproduced in the S1 File as they were originally provided as.eps files, which were not conveniently viewable for many readers.

S1 File can be viewed below.

## Supporting information

S1 File Resolving fluorescent species by their brightness and diffusion using cPCH. (PDF)
