# Supplementary material for: Resolving fluorescent species by their brightness and diffusion using correlated photon-counting histograms
Source: PLoS One. 2019 Dec 30;14(12):e0226063. doi: 10.1371/journal.pone.0226063 (PMC6936799; doi:10.1371/journal.pone.0226063)
Supplement: S1 Table — Both triplet and diffusive binning are necessary. In the third column, the minimum bin size was T = 200 ns and the maximum was T = 3.2 μs. In the fourth column, the minimum bin size was T = 1 μs and the maximum was T = 64 μs. * Both diffusion and triplet binning included in the fit. ** Only diffusion binning included in the fit. R denotes the triplet fraction; τt denotes the triplet relaxation time. (PDF) [file pone.0226063.s009.pdf]

**Parameter**	**Actual Value**	**Fit Value (fit/actual)*** $T_{\min} = 200ns$ $T_{\max} = 3.2\mu$s	**(fit/actual)*** $T_{\min} = 1\mu$s $T_{\max} = 64\mu$s	**(fit/actual)**** $T_{\min} = 1\mu$s $T_{\max} = 64\mu$s
N	0.085	0.084 ±0.0004 (0.988)	(0.995)	(1.147)
ϵ	97791 counts/s	97702 ±377 counts/s (0.999)	(1.004)	(0.867)
τ_d	43.924 μs	41.591 ± 1.131 μs (0.947)	(1.030)	(0.757)
s	1.9276	2.084 ± 0.130 (1.081)	(0.943)	(2.962)
γ_3	0.192	0.199 ± 0.003 (1.037)	(1.032)	(0.998)
γ_4	0.125	0.136 ±0.006 (1.090)	(1.106)	(1.035)
R	0.2	0.195 ±0.004 (0.973)	(1.012)	(1.008)
τ_t	3 μs	3.09 ± 0.19 μs (1.030)	(1.066)	(1.463)

Table S1. Summary of the simulated calibration results including dead-time effects, triplet states, and binning effects. Both triplet and diffusive binning are necessary. In the third column, the minimum bin size was $T = 200$ ns and the maximum was $T = 3.2$ μs. In the fourth column, the minimum bin size was $T = 1$ μs and the maximum was $T = 64$ μs. * Both diffusion and triplet binning included in the fit. ** Only diffusion binning included in the fit. R denotes the triplet fraction; τ_t denotes the triplet relaxation time.
