# Supplementary material for: Correction: Resolving fluorescent species by their brightness and diffusion using correlated photon-counting histograms
Source: PLoS One. 2025 Mar 18;20(3):e0320511. doi: 10.1371/journal.pone.0320511 (PMC11918340; doi:10.1371/journal.pone.0320511)
Supplement: S1 File — (PDF) [file pone.0320511.s001.pdf]

Supporting Information: Resolving fluorescent species by their brightness and diffusion using cPCH

Nathan Scales[1] and Peter S. Swain[2]

[1]Department of Physiology, McGill University, Canada
[2]School of Biological Sciences, University of Edinburgh, United Kingdom

S1 Relating the different bivariate moments

It is simplest to calculate the raw moments from the experimental distribution, using

$$M_{m,n}(\tau) = \sum_{n_x=0}^{\infty}\sum_{n_y=0}^{\infty} {n_x}^m {n_y}^n p_{N_{em}}(n_x, n_y|\tau) \tag{S1}$$

To relate theoretical predictions with experimental results, we typically convert the empirical moments to factorial cumulants because the factorial cumulants have simple expressions and we can use these expressions to find initial parameters in our fitting routines. We will, however, present formulas to allow moments to be converted in either direction, using the cumulants as an intermediary step.

We note that the cumulants of the N-molecule distribution, $K_{m,n}$, are N times the raw moments of the single molecule distribution and that the factorial cumulants of the N-molecule distribution, $(K)_{m,n}$, are N times the factorial moments of the single molecule distribution.

The raw moments and the factorial moments of the single molecule distributions can be related to each other through a sum over the Stirling numbers of the second kind, $S_2(m,n)$ [1, 2], which allows us to express the cumulants in terms of the factorial cumulants:

$$K_{m,n} = \begin{cases} \sum_{p=1}^{m}\sum_{q=1}^{n} S_2(m,p)S_2(n,q)(K)_{p,q} & m>0\ ;\ n>0 \\ \sum_{p=1}^{m} S_2(m,p)(K)_{p,0} & m>0\ ;\ n=0 \\ \sum_{q=1}^{n} S_2(n,q)(K)_{0,q} & m=0\ ;\ n>0 \\ 1 & m=0\ ;\ n=0 \end{cases} \tag{S2}$$

To relate the factorial cumulants, $(K)_{m,n}$, to the cumulants, $K_{m,n}$, we need a Stirling number of the first kind, $s_1(m,n)$:

$$(K)_{m,n} = \begin{cases} \sum_{p=1}^{m}\sum_{q=1}^{n} s_1(m,p)s_1(n,q)K_{p,q} & m>0\ ;\ n>0 \\ \sum_{p=1}^{m} s_1(m,p)K_{p,0} & m>0\ ;\ n=0 \\ \sum_{q=1}^{n} s_1(n,q)K_{0,q} & m=0\ ;\ n>0 \\ 1 & m=0\ ;\ n=0 \end{cases} \tag{S3}$$

To calculate the cumulants, $K_{m,n}$, from the moments, $M_{m,n}$, we can derive a recursion through repeated use of the Leibniz rule for higher order differentiation [2] on the moment generating function, giving:

$$M_{m,n} = \begin{cases} \sum_{p=0}^{m-1}\sum_{q=0}^{n} \binom{m-1}{p}\binom{n}{q} M_{m-1-p,n-q}K_{p+1,q} & m>0 \\ \sum_{q=0}^{n-1} \binom{n-1}{q} M_{0,n-1-q}K_{0,q+1} & m=0\ ;\ n>0 \\ 1 & m=0\ ;\ n=0. \end{cases} \tag{S4}$$

Eq. S4 can be inverted, so we can also relate the cumulants, $K_{m,n}$, to the moments, $M_{m,n}$:

$$K_{m,n} = \begin{cases} M_{m,n} - \sum_{p=0}^{m-1} \sum_{q=0}^{n-1} \binom{m-1}{p} \binom{n}{q} M_{m-1-p,n-q} K_{p+1,q} & \\ \qquad - \sum_{p=0}^{m-2} \binom{m-1}{p} M_{m-1-p,0} K_{p+1,n} & m > 0 \; ; \; n > 0 \\ M_{m,0} - \sum_{p=1}^{m-1} \binom{m-1}{p-1} M_{m-p,0} K_{p,0} & m > 0 \; ; \; n = 0 \\ M_{0,n} - \sum_{q=1}^{n-1} \binom{n-1}{q-1} M_{0,n-q} K_{0,q} & m = 0 \; ; \; n > 0 \\ 1 & m = 0 \; ; \; n = 0 \end{cases} \tag{S5}$$

Reiterating, we wish to convert the empirical raw moments into factorial cumulants so that we can compare theoretical predictions with data. We do so using Eq. S1 to find $M_{m,n}$, Eq. S5 to find $K_{m,n}$, and Eq. S3 to find $(K)_{m,n}$.

The relations here are general and are also used to calculate the expected variance of each moment using the moments of moments (Eq. 36and Sec. S9).

Finally, we note that the Stirling numbers are most easily calculated using recurrence relations [2]:

$$S_2(p, k) = S_2(p-1, k-1) + k S_2(p-1, k) \tag{S6}$$

with $S_2(p, p) = 1$ and $S_2(p, 1) = 1$, and

$$s_1(p, k) = (p-1) s_1(p-1, k) + s_1(p-1, k-1) \tag{S7}$$

with $s_1(0, 0) = 1$, $s_1(p, p) = 1$, and $s_1(p, 0) = 0$ for $p > 0$.

S2 Examples of sampling using analytical inversions of the factorial cumulants

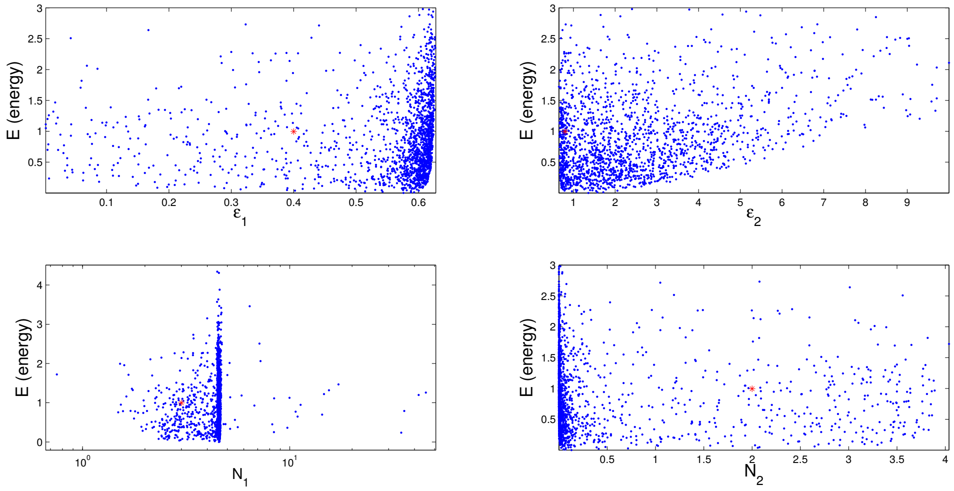

Figure S1. There are many solutions that have energies low enough to be considered within the experimental uncertainty of the data. Scatter plots of the energy of physically valid solutions sampled from inverting the factorial cumulants for a simulation of a mixture of monomers and dimers with $N_1 = 3$, $\epsilon_1 = 0.4$, $N_2 = 2$, and $\epsilon_2 = 2\epsilon_1$. Each blue dot represents the energy corresponding to a sampled solution; the red asterisk shows the energy corresponding to the actual parameters.

Sampling using Eqs. 61 – 62 shows that multiple values of N_1, ϵ_1, N_2, and ϵ_2 are consistent with the data (Fig. S1). To restrict parameter space further, cPCH could be used (by taking

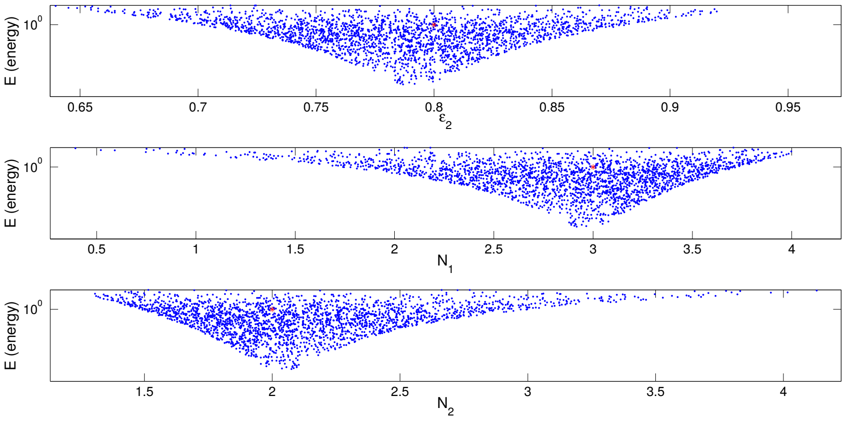

Figure S2. By measuring ϵ_1 *a priori*, there is a minimum in the energy as a function of the parameter values. Scatter plots of the energy for physically valid samples for the same data of Fig. S1.

advantage of the diffusion properties, there will be a reduced dependence on the fourth order moments). Alternatively, another practical option is to use more control experiments. For example, if we have *a priori* knowledge of the brightness of one of the species, ϵ_1 say, then we can sample N_1, ϵ_2, and N_2 from Eqs. 61 – 62 using only empirical measurements of the first three factorial cumulants (Fig. S2). There is now an energy well with its minimum near the true values used in the simulations.

S3 Spatial cPCH

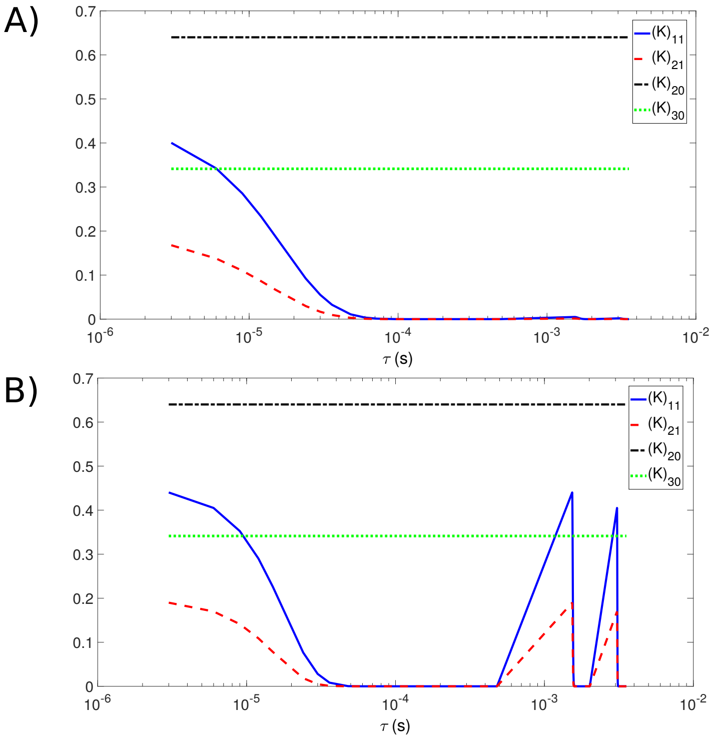

Figure S3. As immobile and mobile fluorophores show different decays in their joint factorial cumulants, spatial cPCH can distinguish one from the other. Plots of the higher order spatial factorial cumulants for a mobile (A) and an immobile species (B). Only the diffusion coefficients have different values.

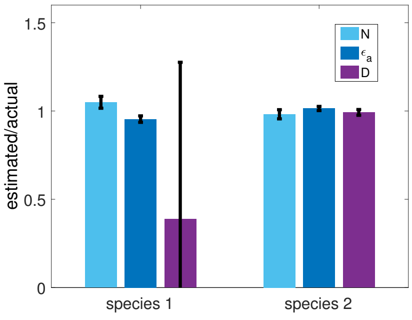

Figure S4. Spatial cPCH can resolve two species with different diffusion coefficients. Inference using a spatial cPCH fit of the factorial cumulants of simulated imaging data using two sets of 25 frames each and with a quickly and a slowly diffusing species ($\epsilon_1 = 31181$ counts/s, $N_1 = 1.2$, $D_1 = 0.1\ \mu\text{m}^2/\text{s}$, $\epsilon_2 = 95452$ counts/s, $N_2 = 0.6$, and $D_2 = 200\ \mu\text{m}^2/\text{s}$). The fitting algorithm only considered τ up to 0.01 s and so was unable to resolve D_1 because $\tau_1 \approx 0.176$ s.

S4 Comparing cPCH with PCH, FCA, and FCS

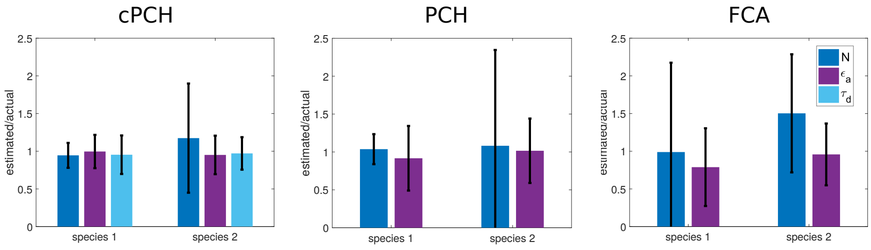

Figure S5. cPCH can outperform alternative methods: a mixture of fluorophores, with one species twice as bright as the other. We simulated data for 240 s and show the ratios of the fit results to the true parameters ($N_1 = 8$, $\epsilon_1 = 48895$ counts/s, $\tau_1 = 175.7$ μs, $N_2 = 2$, $\epsilon_2 = 2\epsilon_1$, and $\tau_2 = 351.39$ μs and a bin time of 10 μs).

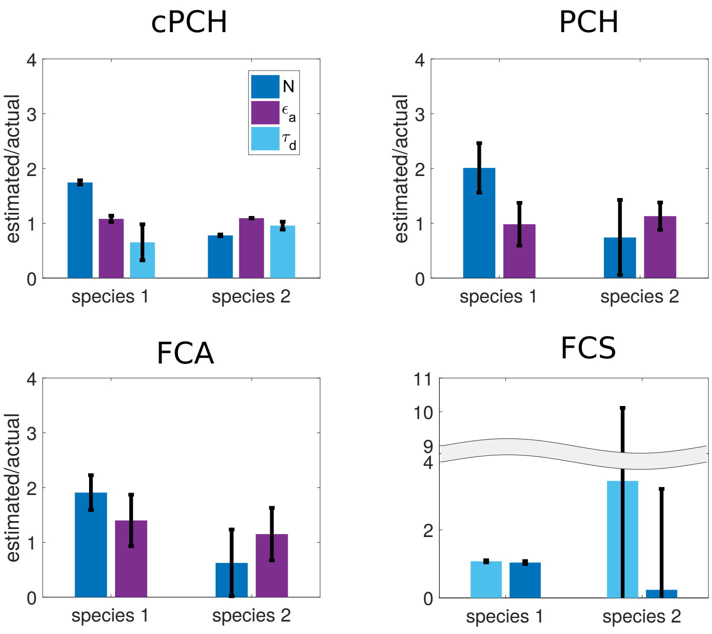

Figure S6. cPCH can out perform alternative methods: two dim species. Analysis of data for a mixture of two dim species, with the brighter species outnumbering the dimmer species, simulated for 210 s. The ratio of the fit results to the actual parameters are shown (N_1 = 0.4, $\epsilon_1 = 14669$ counts/s, $\tau_1 = 175.7$ μs, $N_2 = 0.9$, $\epsilon_2 = 39116$ counts/s, $\tau_2 = 43.9$ μs and a bin time of $T = 10$ μs).

S5 Using cPCH to study ligand binding to a receptor with two distinct binding sites

A challenging problem for FFS is to estimate the dissociation constants of binding of a ligand to a receptor. For example, CFTR, a chloride channel that when mutated can give rise to cystic fibrosis, binds ATP on two distinct binding sites, and the dissociation constants of binding for either are unknown [3].

To demonstrate that the cPCH can in principle estimate these dissociation constants, we will generalize and denote the free ligand as L, an unbound receptor as R, a receptor binding

ligand at one site as R_{01}, a receptor binding ligand at the other site as R_{10}, and a receptor binding ligand at both sites as R_{11}. We assume that the system is at equilibrium and that the dissociation constant of each site is independent of the other — each is unchanged if the other site is bound by ligand.

The ligand, but not the receptor, has a fluorescent tag, and consequently we cannot distinguish R_{01} and R_{10} by either fluorescence or diffusion and instead see an amalgamated state: $R_1 = R_{01} + R_{10}$. If $K_{01} = \frac{LR}{R_{01}}$ and $K_{10} = \frac{LR}{R_{10}}$ are the dissociation constants for the two binding states, then we see effective dissociation constants $K_1^* = \frac{LR}{R_1}$ and $K_2^* = \frac{LR_1}{R_{11}}$. As each reaction is at equilibrium, we can show that

$$K_1^* = \left(K_{01}^{-1} + K_{10}^{-1}\right)^{-1} \quad ; \quad K_2^* = K_{01} + K_{10} \tag{S8}$$

and so

$$\begin{aligned} K_{01} &= \frac{1}{2}\left(K_2^* - \sqrt{K_2^{*2} - 4K_1^* K_2^*}\right) \\ K_{10} &= \frac{1}{2}\left(K_2^* + \sqrt{K_2^{*2} - 4K_1^* K_2^*}\right). \end{aligned} \tag{S9}$$

Therefore we can estimate K_{01} and K_{10} from the effective dissociation constants K_1^* and K_2^*.

Experimentally, we mix an initial amount of fluorescent ligand L_0 with an initial amount of the receptor R_0, allow the system to equilibrate, and measure fluorescence. Without tagged receptors, we must know R_0 to estimate R from $R_0 = R + R_1 + R_{11}$.

To validate the cPCH approach, we fit the factorial cumulants to simulated data. We assume that γ_3, γ_4, and r_A and z_A, the beam-waist parameters of the observation volume, are known so that the number of molecules of each species can be converted to concentrations. There are two sets of species with different brightness: N_1 consisting of the numbers of molecules of L and R_1, and N_{11} consisting of the number of molecules of R_{11}. If R_{11} is twice as bright as R_1, we can solve for N_1 and N_{11} in terms of the factorial cumulants:

$$\begin{aligned} \frac{N_1}{L_0} &= 2 - \frac{\gamma_1^2 (K_2) L_0}{\gamma_2 (K)_1{}^2} \\ \frac{N_{11}}{L_0} &= \frac{\gamma_1{}^2 (K_2) L_0 - \gamma_2 (K)_1{}^2}{2\gamma_2 (K)_1{}^2} \end{aligned} \tag{S10}$$

with ϵ, the brightness of the fluorescent ligand, satisfying

$$\epsilon = \frac{(K)_1}{\gamma_1 L_0}. \tag{S11}$$

We sample the factorial cumulants using their estimated variances (Methods) and then find $(K)_{11}(\tau)$, given by

$$(K)_{11}(\tau) = \gamma_{11}\left[N_L \epsilon^2 g(\tau|\tau_L) + (N_1 - N_L)\epsilon^2 g(\tau|\tau_R) + N_{11}(2\epsilon)^2 g(\tau|\tau_R)\right], \tag{S12}$$

for a range of values of τ_L and τ_R, the diffusion times of the ligand and receptors (assuming that the binding of ligand to the receptor does not change its diffusion), and N_L, the equilibrium numbers of free ligand. To start the nested sampling (Methods), we use values of N_L, N_1, N_{11}, ϵ, τ_L, and τ_R for which the energy of $(K)_{11}(\tau)$ is at a minimum. If the initial concentration of L_0 is unknown, the first three factorial cumulants can be used to estimate N_1, N_{11}, and ϵ, resulting, however, in larger uncertainties in the starting values for the nested sampling.

S6 Triplet states, blinking and dark states

S6.1 Correcting cPCH for dark states

Triplet states, blinking, and other transient dark states can impact photon counts. Focusing on triplet states – other dark states are analysed similarly, we assume that diffusion and triplet effects are independent because they occur on different time scales (a few versus tens to hundreds of micro-seconds) [4]. We will also assume that the probability of entering a triplet state is independent the fluorophore's position in the observation volume.

The single molecule probability, $p_1(n_x, n_y, |\tau)$, is then

$$p_1(n_x, n_y, |\tau) \quad = \quad p(A|\tau) \int \int p_1(n_x, n_y, \overline{x}, \overline{y}|\tau) d\overline{x} d\overline{y} \tag{S13}$$

where $p(A|\tau)$ is the probability of a single molecule being in the fluorescent state A at time τ,

$$p(A|\tau) = 1 - R\left(1 - e^{-\frac{\tau}{\tau_t}}\right) \tag{S14}$$

with R being the triplet fraction, or average occupancy of the triplet state, and τ_t being the triplet lifetime.

The expressions for the factorial cumulants and the cPCH probabilities therefore become multiplied by $p(A|\tau)$, and the N_i for each species becomes multiplied by its fluorescent fraction: $1 - R_i$. For example, the factorial cumulants for cPCH for a single species in a single-channel experiment, Eq. 33 with Eq. 9, would become

$$(K)_{m,n} = N(1 - R){\epsilon_A}^{m+n} \kappa_{m,n}(\tau, D) p_{m,n}(A|\tau) \gamma_{m,n} \tag{S15}$$

with

$$p_{m,n}(A|\tau) = \begin{cases} 1 & \text{for } m = 0 \text{ or } n = 0 \\ p(A|\tau) & \text{for } m > 0 \text{ and } n > 0 \end{cases} \tag{S16}$$

so that the triplet probabilities $p(A|\tau)$ are not introduced into the one-dimensional moments ($m = 0$ or $n = 0$).

The cPCH single-molecule probability, Eq. 6, can be written as

$$p_1(n_x, n_y|\tau) \quad = \quad \frac{V_{PSF_{ab}}}{V} \sum_{k=0}^{\infty} \frac{(-1)^k}{n_x! n_y! k!} \sum_{m=0}^{k} \binom{k}{m} G_{n_x+k-m, n_y+m}(\tau) p_{n_x+k-m, n_y+m}(A|\tau). \tag{S17}$$

S6.2 Binning with triplet states for cPCH

Dark states typically alter the binning functions if the bin time T approaches the relaxation time of the dark state. Following FIMDA [5], we integrate over the triplet correlation function rather than the diffusion correlation function to derive the binning function:

$$B_2(T, \tau_t) \quad = \quad \frac{2}{T^2} \int_{t_1}^{T} \int_{0}^{t_1} p(A|\tau = t_2 - t_1) dt_1 dt_2 \tag{S18}$$

which can be written in terms of the triplet fraction R and the triplet relaxation time τ_t as

$$B_2(T, \tau_t) = 2\left[\left(e^{-\frac{T}{\tau_t}} - 1\right) R \left(\frac{\tau_t}{T}\right)^2 + R\frac{\tau_t}{T} + \frac{1-R}{2}\right]. \tag{S19}$$

Therefore, if the bin time T is much smaller than the triplet relaxation time τ_t, the binning function approaches 1; if $T \gg \tau_t$, the binning function approaches a minimum of $1 - R$.

Assuming independence of diffusion and triplet effects, we can multiply Eq. S19 by the FIMDA binning function, Eq. 46, so that Eq. 49 becomes

$$(K)_n(T) = N\,(\epsilon_o T)^n \left(B_2(T,\tau_d) B_2(T,\tau_t) \right)^{n-1} \gamma_n \tag{S20}$$

and Eq. 50 becomes

$$B_{m,n}(T,\tau_d,\tau_t) \approx \begin{cases} B_2(T,\tau_A)^{m-1} B_2(T,\tau_t)^{m-1}, & n=0 \\ B_2(T,\tau_B)^{n-1} B_2(T,\tau_t)^{n-1}, & m=0 \\ B_2(T,\tau_A)^{m-1} B_2(T,\tau_B)^{n-1} B_2(T,\tau_t)^{m+n-2}, & m>0 \text{ and } n>0 \end{cases} \tag{S21}$$

with $\tau_d = \tau_A$ for channel A and $\tau_d = \tau_B$ for channel B.

S7 Corrections for detector dead-times

A common artifact of detectors is dead-time – the period of time after a detector detects a photon during which it is unable to detect another, which can give underestimates of the number of counts in a given time bin, at least at higher count rates.

For cPCH, we use an established method [6], which corrects the empirical probability distributions of photon counts, denoted $p^*(n_A, n_B)$, for dead-time effects. Let the ratio of the dead-time to the bin size, T, be τ_{dead}. First, we obtain $p^1(n_A, n_B)$ by

$$\begin{aligned} p^1(n_A,n_B) &= p^*(n_A,n_B) - \tau_{\text{dead}} \Big[n_A(n_A+1)p^*(n_A+1,n_B) + n_B(n_B+1)p^*(n_A,n_B+1) \\ &\quad - n_A(n_A-1)p^*(n_A,n_B) - n_B(n_B-1)p^*(n_A,n_B) \Big]. \end{aligned} \tag{S22}$$

Second, we perform a further correction to give $p^{2a}(n_A, n_B)$ and $p^{2b}(n_A, n_B)$:

$$\begin{aligned} p^{2a}(n_A,n_B) &= p^*(n_A,n_B) - \tau_{\text{dead}} \Big[n_A(n_A+1)p^1(n_A+1,n_B) + n_B(n_B+1)p^1(n_A,n_B+1) \\ &\quad - n_A(n_A-1)p^1(n_A,n_B) - n_B(n_B-1)p^1(n_A,n_B) \Big] \end{aligned} \tag{S23}$$

and

$$\begin{aligned} p^{2b}(n_A,n_B) &= -\frac{\tau_{\text{dead}}^2}{2} \Big[{n_A}^2(n_A+1)(n_A+2)p^*(n_A+2,n_B) - \\ &\quad n_A(n_A+1)(2{n_A}^2 - 2n_A + 1)p^*(n_A+1,n_B) + \\ &\quad {n_B}^2(n_B+1)(n_B+2)p^*(n_A,n_B+2) - \\ &\quad n_B(n_B+1)(2{n_B}^2 - 2n_B + 1)p^*(n_A,n_B+1) + \\ &\quad n_A(n_A-1)^3 p^*(n_A,n_B) + n_B(n_B-1)^3 p^*(n_A,n_B) + \\ &\quad 2n_A n_B(n_A+1)(n_B+1)p^*(n_A+1,n_B+1) + \\ &\quad 2n_A n_B(n_A-1)(n_B-1)p^*(n_A,n_B) - \\ &\quad 2n_A n_B(n_A+1)(n_B-1)p^*(n_A+1,n_B) - \\ &\quad 2n_A n_B(n_A-1)(n_B+1)p^*(n_A,n_B+1) \Big] \end{aligned} \tag{S24}$$

so that our final corrected distribution, $p(n_A, n_B)$, obeys

$$p(n_A,n_B) = p^{2a}(n_A,n_B) + p^{2b}(n_A,n_B). \tag{S25}$$

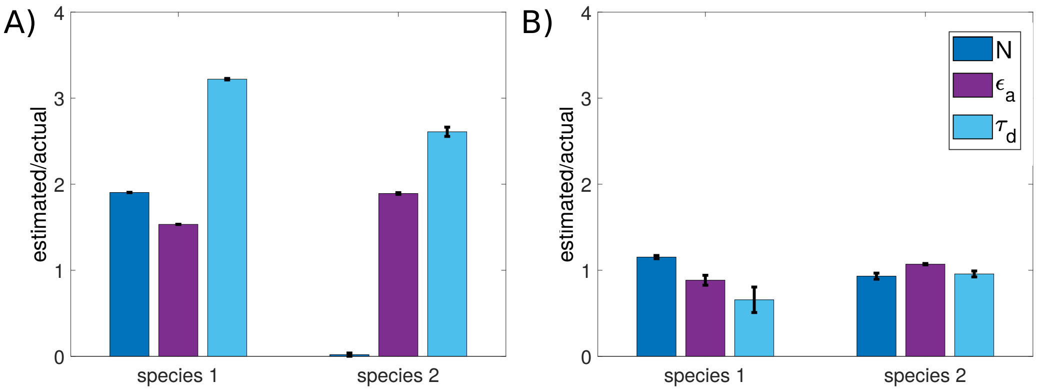

H

Figure S7. Dead-time corrections are necessary when dead-time is present. A nested sampling fit to a MesoRD simulation employing a 50 ns dead-time: **A)** without or **B)** with dead-time corrections. For this simulation, $N_1 = 0.213$, $\epsilon_1 = 88900$ counts/s, and $\tau_1 = 43.92$ μs for species 1, and $N_2 = 0.213$, $\epsilon_2 = 177800$ counts/s, and $\tau_2 = 175.7$ μs for species 2. The maximum bin size is $T = 3.2$ μs and the detector dead-time was 50ns.

We tested this approach using simulations and in this example we can resolve two different species only if dead-time is corrected (Fig. S7). The MesoRD simulator was run with a bin size of 50 ns in the mode where it reports both arrival times to the nearest 50 ns and the number of photons that arrived within that 50 ns window. To mimic a detector with dead-time, we ignored the number of photons detected within the 50 ns and treated each arrival time instead as a single photon detection. We then used 2000 analytical solutions to the resampled factorial cumulant equations as initial conditions to a nested sampling run with 200 objects to fit the cPCH factorial cumulants both with and without correcting for dead-time.

S7.1 Verifying corrections for triplet effects and detector dead-time

We verified our corrections for triplet effects and the detector dead-time using simulations (Table 1). Using MesoRD, we simulated a single species, and in addition to the usual parameters, such as the brightness, concentration, and diffusion time, we also estimated the shape factors γ_3 and γ_4, the structure factor s, as well as the triplet fraction R and relaxation time τ_t.

**Parameter**	**Actual Value**	**Fit Value (fit/actual)*** $T_{\min} = 200ns$ $T_{\max} = 3.2\mu$s	**(fit/actual)*** $T_{\min} = 1\mu$s $T_{\max} = 64\mu$s	**(fit/actual)**** $T_{\min} = 1\mu$s $T_{\max} = 64\mu$s
N	0.085	0.084 ±0.0004 (0.988)	(0.995)	(1.147)
ϵ	97791 counts/s	97702 ±377 counts/s (0.999)	(1.004)	(0.867)
τ_d	43.924 μs	41.591 ± 1.131 μs (0.947)	(1.030)	(0.757)
s	1.9276	2.084 ± 0.130 (1.081)	(0.943)	(2.962)
γ_3	0.192	0.199 ± 0.003 (1.037)	(1.032)	(0.998)
γ_4	0.125	0.136 ±0.006 (1.090)	(1.106)	(1.035)
R	0.2	0.195 ±0.004 (0.973)	(1.012)	(1.008)
τ_t	3 μs	3.09 ± 0.19 μs (1.030)	(1.066)	(1.463)

Table 1. Summary of the simulated calibration results including dead-time effects, triplet states, and binning effects. Both triplet and diffusive binning are necessary. In the third column, the minimum bin size was $T = 200$ ns and the maximum was $T = 3.2$ μs. In the fourth column, the minimum bin size was $T = 1$ μs and the maximum was $T = 64$ μs. * Both diffusion and triplet binning included in the fit. ** Only diffusion binning included in the fit. R denotes the triplet fraction; τ_t denotes the triplet relaxation time.

S8 Corrections for detector afterpulsing

Photon counting detectors have a low probability of generating an afterpulse after the detection of a photon, which cannot be distinguished from a real photon and alters the statistics of the detected light signal. A challenge in correcting for afterpulses is to correct for afterpulses that originated in different time bins – the afterpulse occurs in a later time bin than the original photon.

We will correct for afterpulsing by correcting the experimental distribution, rather than modifying its analysis; however in the derivations, we provide equations to allow the theoretical distributions to be corrected instead if desired.

S8.1 Afterpulsing for single channel data

Let $p(n, k|q)$ be the probability of detecting n photons and k afterpulses if the probability of generating an afterpulse is q, which obeys

$$p(n, k|q) = p(k|n, q)p(n) \tag{S26}$$

where $p(k|n, q)$ is the probability of detecting k afterpulses given that we detected n photons and $p(n)$ is the probability of detecting n photons without afterpulsing.

If each photon can only produce one afterpulse, the probability $p(k|n, q)$ is given by the binomial distribution:

$$p(k|n, q) = p_{\text{bin}}(k|n, q) \equiv \binom{n}{k} q^k (1-q)^{n-k}. \tag{S27}$$

Experimentally, we measure the probability of detecting n pulses in total, $p^*(n|q)$, some of which may or may not be afterpulses:

$$\begin{aligned} p^*(n|q) &= \sum_{k=0}^{n} p(n-k, k|q) \\ &= \sum_{k=0}^{n} \binom{n-k}{k} q^k (1-q)^{n-2k} p(n-k) \end{aligned} \tag{S28}$$

To correct our distribution, we rearrange to find $p(n)$, which by definition has no afterpulses:

$$p(n) = \frac{p^*(n|q) - \sum_{k=1}^{n} \binom{n-k}{k} q^k (1-q)^{n-2k} p(n-k)}{(1-q)^n} \tag{S29}$$

Eq. S29 corrects a single channel distribution, but we need to extend this result to two channels for cPCH.

S8.2 Afterpulsing for two channels

Let the detectors for the two channels have afterpulsing probabilities of q_A and q_B. Afterpulses in one detector are assumed to be uncorrelated with afterpulses in the other. We define the probability of detecting n_A counts in channel A and n_B counts in channel B with k_A afterpulses in channel A and k_B afterpulses in channel B as $p(n_A, n_B, k_A, k_B|q_A, q_B)$. As before

$$p(n_A, n_B, k_A, k_B|q_A, q_B) = p_{\text{bin}}(k_A|n_A, q_B) p_{\text{bin}}(k_B|n_B, q_B) p(n_A, n_B). \tag{S30}$$

and

$$p^*(n_A, n_B|q_A, q_B) = \sum_{k_A=0}^{n_A} \sum_{k_B=0}^{n_B} p_{\text{bin}}(k_A|n_A - k_A, q_A) p_{\text{bin}}(k_B|n_B - k_B, q_B) p(n_A - k_A, n_B - k_B). \tag{S31}$$

Again, we solve for $p(n_A, n_B)$

$$\begin{aligned} p(n_A, n_B)(1-q_A)^{n_A}(1-q_B)^{n_B} \;=\; & p^*(n_A, n_B|q_A, q_B) - \sum_{k_A=1}^{n_A} \sum_{k_B=0}^{n_B} p_{\text{bin}}(k_A|n_A - k_A, q_A) \\ & \times p_{\text{bin}}(k_B|n_B - k_B, q_B) p(n_A - k_A, n_B - k_B) \\ & + \sum_{k_B=1}^{n_B} (1-q_A)^{n_A} p_{\text{bin}}(k_B|n_B - k_B, q_B) p(n_A, n_B - k_B). \end{aligned} \tag{S32}$$

Once the afterpulse probabilities q_a and q_b have been measured [7], Eq. S32 can in principle be used to correct $p^*(n_A, n_B|q_A, q_B)$ for afterpulses in the same time bin; however, modifications to the theory are required if we wish to correct for afterpulses across bins.

S8.3 Afterpulsing in correlated time bins

The afterpulse probability q of a detector is the probability of obtaining an afterpulse, $p(\tau)$, at any time τ, after the original photon detection. The afterpulse probability q_T for a time bin of duration T is $q_T = \int_0^T p(\tau) d\tau$.

Consider $p(n_A, n_B, k_A, k_B, k_\tau|\tau, p(\tau))$ – the probability of detecting n_A photons and k_A afterpulses at time 0 and of detecting n_B photons and both k_B afterpulses at time τ, where the afterpulses originate in this time bin, and k_τ afterpulses that originated in time bin 0. Therefore $T < \tau$ and $q_A = q_B = q_T$ because afterpulses can only occur in the same detector.

Factorizing this probability, assuming that afterpulses generated from different time bins are independent of afterpulses generated within the same time bin:

$$p(n_A, n_B, k_A, k_B, k_\tau|\tau, p(\tau)) \;=\; p(k_\tau|n_A, n_B, p(\tau), \tau) p(k_A, k_B|n_A, n_B, q_T) p(n_A, n_B|\tau) \tag{S33}$$

where $p(n_A, n_B|\tau)$ is the cPCH distribution. The term $p(k_A, k_B|n_A, n_B, q_T)$ can be decomposed into binomial probabilities:

$$p(k_A, k_B|n_A, n_B, q_T) = p_{\text{bin}}(k_A|n_A, q_T) p_{\text{bin}}(k_B|n_B, q_T). \tag{S34}$$

To relate Eq. S33 to the empirical distribution, $p^*(n_a, n_b|\tau, p(\tau))$, we consider first

$$\begin{aligned} p^*(n_A, n_B, k_\tau|\tau, p(\tau)) &= \sum_{k_A=0}^{n_A} \sum_{k_B=0}^{n_B} p(k_\tau|n_A, n_B, p(\tau), \tau) p_{\rm bin}(k_A|n_A - k_A, q_T) \\ &\quad \times p_{\rm bin}(k_B|n_B - k_B, q_T) p(n_A - k_A, n_B - k_B|\tau) \end{aligned} \tag{S35}$$

To include afterpulsing across different time bins, we need to sum Eq. S35 over k_τ. The k_τ afterpulses can only occur because of the $n_A - k_A$ photons that occurred at time 0 in Eq. S35. The probability of generating k_τ afterpulses from $n_A - k_A$ photons is binomial with probability $p(\tau)$: $p_{\rm bin}(k_\tau|n_A - k_A, p(\tau))$. A final complication is that the n_B pulses at time τ are generated not only from the k_τ pulses but also from pulses generated within the time bin. Consequently,

$$\begin{aligned} p^*(n_A, n_B|\tau, p(\tau)) &= \sum_{k_A=0}^{n_A} \sum_{k_\tau=0}^{\min\{n_A-k_A, n_B\}} \sum_{k_B=0}^{n_B-k_\tau} p_{\rm bin}(k_\tau|n_A - k_A, p(\tau)) \\ &\quad \times p_{\rm bin}(k_A|n_A - k_A, q_T) p_{\rm bin}(k_B|n_B - k_B - k_\tau, q_T) \\ &\quad \times p(n_A - k_A, n_B - k_B - k_\tau|\tau). \end{aligned} \tag{S36}$$

As before, we rearrange to find $p(n_A, n_B|\tau)$:

$$\begin{aligned} p(n_A, n_B|\tau)(1 - q_T)^{2n_A+n_B} &= p^*(n_A, n_B|\tau, p(\tau)) \\ &\quad - \sum_{k_A=1}^{n_A} \sum_{k_\tau=0}^{\min\{n_A-k_A, n_B\}} \sum_{k_B=0}^{n_B-k_\tau} \phi(k_A, k_\tau, k_B) \\ &\quad - \sum_{k_\tau=1}^{\min\{n_A, n_B\}} \sum_{k_B=0}^{n_B-k_\tau} \phi(0, k_\tau, k_B) - \sum_{k_B=1}^{n_B} \phi(0, 0, k_B) \end{aligned} \tag{S37}$$

with

$$\begin{aligned} \phi(k_A, k_\tau, k_B) &= p_{\rm bin}(k_\tau|n_A - k_A, p(\tau)) p_{\rm bin}(k_A|n_A - k_A, q_T) \\ &\quad \times p_{\rm bin}(k_B|n_B - k_B - k_\tau, q_T) p(n_A - k_A, n_B - k_B - k_\tau|\tau). \end{aligned} \tag{S38}$$

Eq. S37 corrects for the effects of afterpulsing that extend beyond a particular time bin and so can remove the large peak seen at short correlation times in empirical FCS curves. Nevertheless, $p(\tau)$ must be known.

To find $p(\tau)$, we modify an existing protocol to measure q_T [7]. First, a control experiment involving a Poisson-distributed light source is required – for example, focusing on a blank coverslip. The count rate should be kept high enough to have reliable signal statistics, but low enough to make any dead-time effects negligible. After a histogram of photon arrival times is generated, we fit with an exponential distribution, appropriate for a Poisson-distributed light source:

$$y(\tau) = A_0 e^{-\tau/\tau_a}. \tag{S39}$$

Second, we isolate the contribution from afterpulses by subtracting the exponential fit from the empirical histogram (Fig. S8A), ignoring the region within the dead-time of the detector. If we define the afterpulse histogram, $x(\tau)$, to be this difference between the empirical histogram, $h(\tau)$, and the exponential fit, $y(\tau)$, so that $x(\tau) = h(\tau) - y(\tau)$, then the probability of afterpulsing, $p(\tau)$, at time τ for a bin width of T can be estimated using

$$p(\tau) = \int_0^{\tau+T/2} x(\tau) d\tau - \int_0^{\tau-T/2} x(\tau) d\tau. \tag{S40}$$

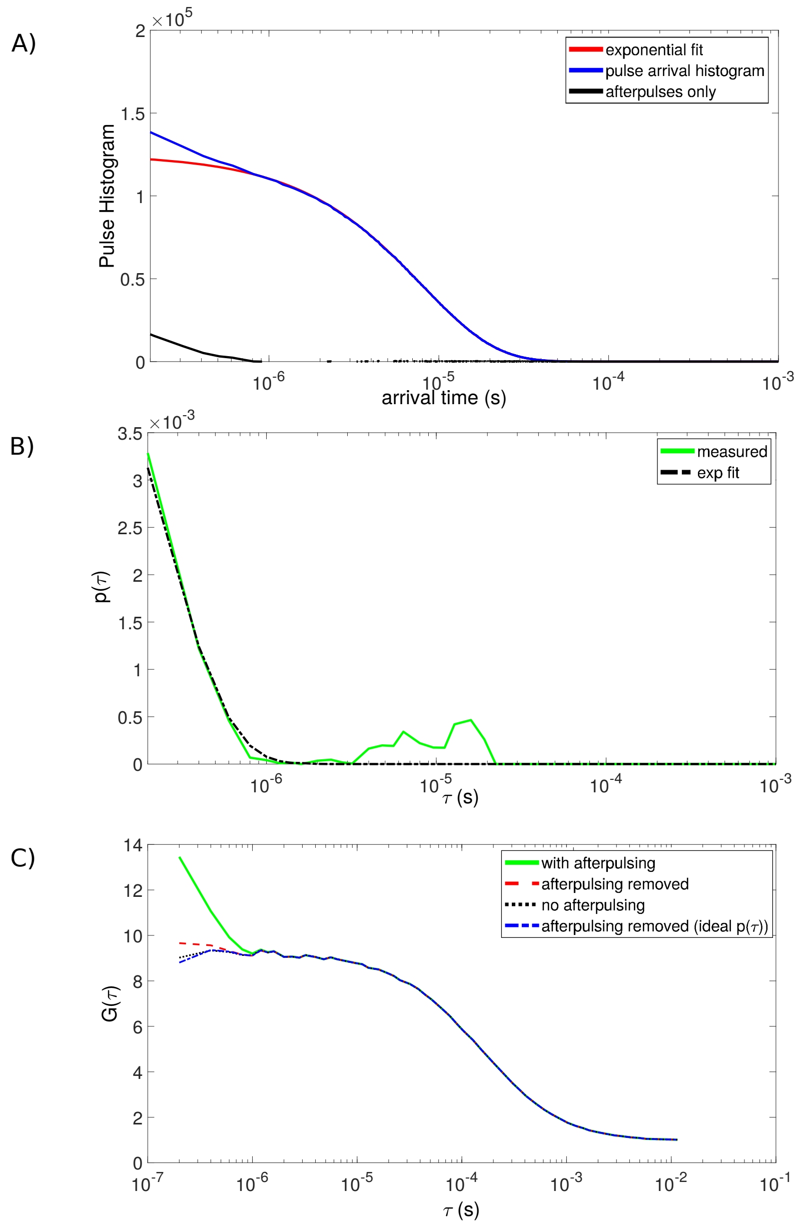

Figure S8. Correcting for afterpulses. Simulated calibration data used to estimate $p(\tau)$ and a demonstration of the removal of afterpulsing effects between time bins for an afterpulse probability $q^* = 0.01$ and an average arrival time $\tau_{\mathrm{ap}} = 200$ ns. **A)** Histograms of arrival times and afterpulses. **B)** Fit to the measured $p(\tau)$. **C)** A comparison of the calculated FCS curves for a 1 nM solution with a diffusion coefficient of 1×10^{-10} m^2/s, with afterpulsing, without afterpulsing, and with afterpulsing removed (using simulated data). The black dotted lines show the FCS curves of the original data without afterpulsing; the green curves show the FCS curves with afterpulsing added to the data; the dashed red curves show the afterpulse-corrected FCS curve using the measured $p(\tau)$; and the blue curves show the afterpulse-corrected FCS curve using the ideal $p(\tau)$.

This estimate of $p(\tau)$ can be noisy at larger values of τ. The afterpulsing probability is expected to be approximately exponential [8], and we fit $x(\tau)$ to an exponential distribution to improve the estimated $p(\tau)$. If τ^* and q^* are parameters from the fit, then

$$p(\tau) = \frac{q^*}{\tau^*}\left[\int_0^{\tau+T/2} \mathrm{e}^{-\frac{s}{\tau^*}} ds - \int_0^{\tau-T/2} \mathrm{e}^{-\frac{s}{\tau^*}} ds\right]. \tag{S41}$$

The resulting $p(\tau)$ can be used in Eq. S37 (Fig. S8B).

S8.4 Verification of afterpulsing in correlated time bins

We verified Eq. S37 using simulations (Fig. S8C). Afterpulses were added to data generated in MesoRD using a dead-time of 50 ns. For each detected photon, we generated an afterpulse with a probability of q^* with its arrival time after the detected photon chosen from an exponential distribution with a rate $\tau_{\text{ap}} = 200$ ns. Afterpulses that occurred within the dead-time of the detector were ignored. We obtained the $p^*(n_a, n_b|\tau)$ distribution from the arrival times and calculated its moments to find the FCS curve. Next, we simulated the experimental measurement of $p(\tau)$ by using an exponential distribution for the arrival times to generate a Poisson-distributed set of photon counts. Afterpulses were added, and the apparent $p(\tau)$ distribution found. With this distribution, we corrected $p^*(n_a, n_b|\tau)$ using Eq. S37 and obtained a corrected FCS curve from its moments.

S9 Expressions for the variance of the factorial cumulants

Following Eq. 36, we implemented the recurrence relations and the variance expressions in Mathematica (Wolfram Research, Champaign, IL) and find, with N_d being the number of data points (the number of bins used):

$$N_d\mathrm{Var}[(K)_{0,1}] = (K)_{0,1} + (K)_{0,2} \tag{S42}$$

$$\begin{aligned} N_d\mathrm{Var}[(K)_{1,1}] &= (K)_{1,1} + (K)_{1,1}^2 + (K)_{1,2} + (K)_{0,1}\{(K)_{1,0} + (K)_{2,0}\} \\ &\quad +(K)_{0,2}\{(K)_{1,0} + (K)_{2,0}\} + (K)_{2,1} + (K)_{2,2} \end{aligned} \tag{S43}$$

$$N_d\mathrm{Var}[(K)_{2,0}] = 2(K)_{1,0}^2 + 2(K)_{2,0} + 4(K)_{1,0}(K)_{2,0} + 2(K)_{2,0}^2 + 4(K)_{3,0} + (K)_{4,0} \tag{S44}$$

$$\begin{aligned} N_d\mathrm{Var}[(K)_{3,0}] &= 6(K)_{1,0}^3 + 18(K)_{1,0}^2(K)_{2,0} + 54(K)_{2,0}^2 + 6(K)_{2,0}^3 + 6(K)_{3,0} \\ &\quad +9(K)_{3,0}^2 + 18(K)_{4,0} + 9(K)_{1,0} \\ &\quad \times\Big\{2(K)_{2,0} + 2(K)_{2,0}^2 + 4(K)_{3,0} + (K)_{4,0}\Big\} + \\ &\quad 9(K)_{2,0}(8(K)_{3,0} + (K)_{4,0}) + 9(K)_{5,0} + (K)_{6,0} \end{aligned} \tag{S45}$$

$$\begin{aligned} N_d\mathrm{Var}[(K)_{2,1}] &= 4(K)_{1,0}(K)_{1,1} + 4(K)_{1,1}^2 + 4(K)_{1,0}(K)_{1,1}^2 + 4(K)_{1,0}(K)_{1,2} \\ &\quad +12(K)_{1,1}(K)_{2,0} + 4(K)_{1,1}^2(K)_{2,0} + 12(K)_{1,2}(K)_{2,0} + 2(K)_{2,1} \\ &\quad +4(K)_{1,0}(K)_{2,1} + 16(K)_{1,1}(K)_{2,1} + 4(K)_{2,0}(K)_{2,1} + 5(K)_{2,1}^2 \\ &\quad +2(K)_{2,2} + 4(K)_{1,0}(K)_{2,2} + 4(K)_{2,0}(K)_{2,2} + 4(K)_{1,1}(K)_{3,0} \\ &\quad +4(K)_{1,2}(K)_{3,0} + 4(K)_{3,1} + 4(K)_{1,1}(K)_{3,1} + 4(K)_{3,2} + \\ &\quad (K)_{0,1}\Big\{2(K)_{1,0}^2 + 2(K)_{2,0} + 4(K)_{1,0}(K)_{2,0} + 2(K)_{2,0}^2 + 4(K)_{3,0} \\ &\quad +(K)_{4,0}\Big\} + (K)_{0,2}\Big\{2(K)_{1,0}^2 + 2(K)_{2,0} + 4(K)_{1,0}(K)_{2,0} \\ &\quad +2(K)_{2,0}^2 + 4(K)_{3,0} + (K)_{4,0}\Big\}(K)_{4,1} + (K)_{4,2} \end{aligned} \tag{S46}$$

$$\begin{aligned} N_d\mathrm{Var}[(K)_{1,2}] &= 4(K)_{0,3}(K)_{1,0} + (K)_{0,4}(K)_{1,0} + 4(K)_{0,3}(K)_{1,1} + 4(K)_{1,1}^2 \\ &\quad +2(K)_{1,2} + 16(K)_{1,1}(K)_{1,2} + 5(K)_{1,2}^2 + 4(K)_{1,3} + 4(K)_{1,1}(K)_{1,3} \\ &\quad +(K)_{1,4} + 4(K)_{0,3}(K)_{2,0} + (K)_{0,4}(K)_{2,0} + 2(K)_{0,1}^2((K)_{1,0} \\ &\quad +(K)_{2,0}) + 2(K)_{0,2}^2((K)_{1,0} + (K)_{2,0}) + 4(K)_{0,3}(K)_{2,1} + 2(K)_{2,2} \\ &\quad +4(K)_{0,1}\Big\{(K)_{1,1} + (K)_{1,1}^2 + (K)_{1,2} + (K)_{0,2}((K)_{1,0} + (K)_{2,0}) \\ &\quad +(K)_{2,1} + (K)_{2,2}\Big\} + 2(K)_{0,2}\Big\{(K)_{1,0} + 6(K)_{1,1} + 2(K)_{1,1}^2 \\ &\quad +2(K)_{1,2} + (K)_{2,0} + 6(K)_{2,1} + 2(K)_{2,2}\Big\} + 4(K)_{2,3} + (K)_{2,4} \end{aligned} \tag{S47}$$

$$
\begin{aligned}
&N_d \mathrm{Var}[(K)_{2,2}] = \\
&\quad 8(K)_{0,3}(K)_{1,0}^2 + 2(K)_{0,4}(K)_{1,0}^2 + 16(K)_{0,3}(K)_{1,0}(K)_{1,1} + 8(K)_{1,1}^2 + 16(K)_{1,0}(K)_{1,1}^2 \\
&\quad +48(K)_{1,1}^3 + 4(K)_{1,1}^4 + 8(K)_{1,0}(K)_{1,2} + 48(K)_{1,1}(K)_{1,2} + 64(K)_{1,0}(K)_{1,1}(K)_{1,2} \\
&\quad +64(K)_{1,1}^2(K)_{1,2} + 12(K)_{1,2}^2 + 20(K)_{1,0}(K)_{1,2}^2 + 16(K)_{1,0}(K)_{1,3} \\
&\quad +16(K)_{1,1}(K)_{1,3} + 16(K)_{1,0}(K)_{1,1}(K)_{1,3} + 4(K)_{1,0}(K)_{1,4} + 8(K)_{0,3}(K)_{2,0} \\
&\quad +2(K)_{0,4}(K)_{2,0} + 16(K)_{0,3}(K)_{1,0}(K)_{2,0} + 4(K)_{0,4}(K)_{1,0}(K)_{2,0} \\
&\quad +32(K)_{0,3}(K)_{1,1}(K)_{2,0} + 16(K)_{1,1}^2(K)_{2,0} + 24(K)_{1,2}(K)_{2,0} + 64(K)_{1,1}(K)_{1,2}(K)_{2,0} \\
&\quad +20(K)_{1,2}^2(K)_{2,0} + 48(K)_{1,3}(K)_{2,0} + 16(K)_{1,1}(K)_{1,3}(K)_{2,0} + 12(K)_{1,4}(K)_{2,0} \\
&\quad +8(K)_{0,3}(K)_{2,0}^2 + 2(K)_{0,4}(K)_{2,0}^2 + 8(K)_{0,3}(K)_{2,1} + 16(K)_{0,3}(K)_{1,0}(K)_{2,1} \\
&\quad +48(K)_{1,1}(K)_{2,1} + 64(K)_{1,1}^2(K)_{2,1} + 144(K)_{1,2}(K)_{2,1} + 64(K)_{1,1}(K)_{1,2}(K)_{2,1} \\
&\quad +48(K)_{1,3}(K)_{2,1} + 16(K)_{0,3}(K)_{2,0}(K)_{2,1} + 12(K)_{2,1}^2 + 4(K)_{2,2} + 8(K)_{1,0}(K)_{2,2} \\
&\quad +112(K)_{1,1}(K)_{2,2} + 20(K)_{1,1}^2(K)_{2,2} + 64(K)_{1,2}(K)_{2,2} + 8(K)_{2,0}(K)_{2,2} \\
&\quad +64(K)_{2,1}(K)_{2,2} + 17(K)_{2,2}^2 + 8(K)_{2,3} + 16(K)_{1,0}(K)_{2,3} + 32(K)_{1,1}(K)_{2,3} \\
&\quad +16(K)_{2,0}(K)_{2,3} + 20(K)_{2,1}(K)_{2,3} + 2(K)_{2,4} + 4(K)_{1,0}(K)_{2,4} + 4(K)_{2,0}(K)_{2,4} \\
&\quad +16(K)_{0,3}(K)_{3,0} + 4(K)_{0,4}(K)_{3,0} + 8(K)_{0,3}(K)_{1,1}(K)_{3,0} + 8(K)_{1,2}(K)_{3,0} \\
&\quad +16(K)_{1,3}(K)_{3,0} + 4(K)_{1,4}(K)_{3,0} + 16(K)_{0,3}(K)_{3,1} + 16(K)_{1,1}(K)_{3,1} \\
&\quad +48(K)_{1,2}(K)_{3,1} + 16(K)_{1,3}(K)_{3,1} + 8(K)_{3,2} + 32(K)_{1,1}(K)_{3,2} + 20(K)_{1,2}(K)_{3,2} \\
&\quad +16(K)_{3,3} + 8(K)_{1,1}(K)_{3,3} + 4(K)_{3,4} + 4(K)_{0,3}(K)_{4,0} + (K)_{0,4}(K)_{4,0} \\
&\quad +2(K)_{0,1}^2\Big\{2(K)_{1,0}^2 + 2(K)_{2,0} + 4(K)_{1,0}(K)_{2,0} + 2(K)_{2,0}^2 + 4(K)_{3,0} + (K)_{4,0}\Big\} \\
&\quad +2(K)_{0,2}^2\Big\{2(K)_{1,0}^2 + 2(K)_{2,0} + 4(K)_{1,0}(K)_{2,0} + 2(K)_{2,0}^2 + 4(K)_{3,0} + (K)_{4,0}\Big\} \\
&\quad +4(K)_{0,3}(K)_{4,1} + 2(K)_{4,2} + 4(K)_{0,1}\Big\{4(K)_{1,1}^2 + 12(K)_{1,1}(K)_{2,0} + 4(K)_{1,1}^2(K)_{2,0} \\
&\quad +12(K)_{1,2}(K)_{2,0} + 2(K)_{2,1} + 16(K)_{1,1}(K)_{2,1} + 4(K)_{2,0}(K)_{2,1} + 5(K)_{2,1}^2 + 2(K)_{2,2} \\
&\quad +4(K)_{2,0}(K)_{2,2} + 4(K)_{1,0}\Big\{(K)_{1,1} + (K)_{1,1}^2 + (K)_{1,2} + (K)_{2,1} + (K)_{2,2}\Big\} \\
&\quad +4(K)_{1,1}(K)_{3,0} + 4(K)_{1,2}(K)_{3,0} + 4(K)_{3,1} + 4(K)_{1,1}(K)_{3,1} + 4(K)_{3,2} \\
&\quad +(K)_{0,2}\Big\{2(K)_{1,0}^2 + 2(K)_{2,0} + 4(K)_{1,0}(K)_{2,0} + 2(K)_{2,0}^2 + 4(K)_{3,0} + (K)_{4,0}\Big\} \\
&\quad +(K)_{4,1} + (K)_{4,2}\Big\} + 2(K)_{0,2}\Big\{2(K)_{1,0}^2 + 2(K)_{2,0} + 24(K)_{1,2}(K)_{2,0} + 2(K)_{2,0}^2 \\
&\quad +8(K)_{1,1}^2(1 + (K)_{2,0}) + 12(K)_{2,1} + 24(K)_{2,0}(K)_{2,1} + 10(K)_{2,1}^2 + 4(K)_{2,2} \\
&\quad +8(K)_{2,0}(K)_{2,2} + 4(K)_{1,0}\Big\{6(K)_{1,1} + 2(K)_{1,1}^2 + 2(K)_{1,2} + (K)_{2,0} + 6(K)_{2,1} \\
&\quad +2(K)_{2,2}\Big\} + 4(K)_{3,0} + 8(K)_{1,2}(K)_{3,0} + 24(K)_{3,1} + 8(K)_{1,1}(7(K)_{2,0} + 4(K)_{2,1} \\
&\quad +2(K)_{3,0} + (K)_{3,1}) + 8(K)_{3,2} + (K)_{4,0} + 6(K)_{4,1} + 2(K)_{4,2}\Big\} + 4(K)_{4,3} \\
&\quad +(K)_{4,4}
\end{aligned}
\tag{S48}
$$

$$
\begin{aligned}
N_d\mathrm{Var}[(K)_{1,3}] \quad = \quad & 6(K)_{0,3}(K)_{1,0} + 9(K)_{0,3}^2(K)_{1,0} + 18(K)_{0,4}(K)_{1,0} + 9(K)_{0,5}(K)_{1,0} + \\
& (K)_{0,6}(K)_{1,0} + 72(K)_{0,3}(K)_{1,1} + 45(K)_{0,4}(K)_{1,1} + 6(K)_{0,5}(K)_{1,1} + \\
& 72(K)_{0,3}(K)_{1,1}^2 + 9(K)_{0,4}(K)_{1,1}^2 + 90(K)_{0,3}(K)_{1,2} + 15(K)_{0,4}(K)_{1,2} + \\
& 36(K)_{1,1}(K)_{1,2} + 54(K)_{0,3}(K)_{1,1}(K)_{1,2} + 108(K)_{1,2}^2 + 6(K)_{1,3} + \\
& 18(K)_{0,3}(K)_{1,3} + 108(K)_{1,1}(K)_{1,3} + 162(K)_{1,2}(K)_{1,3} + 19(K)_{1,3}^2 + \\
& 18(K)_{1,4} + 54(K)_{1,1}(K)_{1,4} + 24(K)_{1,2}(K)_{1,4} + 9(K)_{1,5} + 6(K)_{1,1}(K)_{1,5} + \\
& (K)_{1,6} + 6(K)_{0,3}(K)_{2,0} + 9(K)_{0,3}^2(K)_{2,0} + 18(K)_{0,4}(K)_{2,0} + 9(K)_{0,5}(K)_{2,0} + \\
& (K)_{0,6}(K)_{2,0} + 6(K)_{0,1}^3((K)_{1,0} + (K)_{2,0}) + 6(K)_{0,2}^3((K)_{1,0} + (K)_{2,0}) + \\
& 72(K)_{0,3}(K)_{2,1} + 45(K)_{0,4}(K)_{2,1} + 6(K)_{0,5}(K)_{2,1} + 90(K)_{0,3}(K)_{2,2} + \\
& 15(K)_{0,4}(K)_{2,2} + 18(K)_{0,1}^2\Big\{(K)_{1,1} + (K)_{1,1}^2 + (K)_{1,2} + (K)_{0,2}\Big\{(K)_{1,0} + \\
& (K)_{2,0}\Big\} + (K)_{2,1} + (K)_{2,2}\Big\} + 18(K)_{0,2}^2\Big\{3(K)_{1,0} + 5(K)_{1,1} + (K)_{1,1}^2 + \\
& (K)_{1,2} + 3(K)_{2,0} + 5(K)_{2,1} + (K)_{2,2}\Big\} + 6(K)_{2,3} + 18(K)_{0,3}(K)_{2,3} + \\
& 18(K)_{2,4} + 9(K)_{0,1}\Big\{(K)_{0,4}(K)_{1,0} + 4(K)_{1,1}^2 + 2(K)_{1,2} + 16(K)_{1,1}(K)_{1,2} + \\
& 5(K)_{1,2}^2 + 4(K)_{1,3} + 4(K)_{1,1}(K)_{1,3} + (K)_{1,4} + (K)_{0,4}(K)_{2,0} + \\
& 2(K)_{0,2}^2((K)_{1,0} + (K)_{2,0}) + 4(K)_{0,3}((K)_{1,0} + (K)_{1,1} + (K)_{2,0} + (K)_{2,1}) + \\
& 2(K)_{2,2} + 2(K)_{0,2}\Big\{(K)_{1,0} + 6(K)_{1,1} + 2(K)_{1,1}^2 + 2(K)_{1,2} + (K)_{2,0} + \\
& 6(K)_{2,1} + 2(K)_{2,2}\Big\} + 4(K)_{2,3} + (K)_{2,4}\Big\} + 9(K)_{0,2}\Big\{2(K)_{1,1} + 14(K)_{1,1}^2 + \\
& 12(K)_{1,2} + 28(K)_{1,1}(K)_{1,2} + 5(K)_{1,2}^2 + 8(K)_{1,3} + 4(K)_{1,1}(K)_{1,3} + (K)_{1,4} + \\
& (K)_{0,4}((K)_{1,0} + (K)_{2,0}) + 2(K)_{2,1} + 4(K)_{0,3}\Big\{2(K)_{1,0} + (K)_{1,1} + 2(K)_{2,0} + \\
& (K)_{2,1}\Big\} + 12(K)_{2,2} + 8(K)_{2,3} + (K)_{2,4}\Big\} + 9(K)_{2,5} + (K)_{2,6} \qquad \text{(S49)}
\end{aligned}
$$

The variance $\mathrm{Var}[(K)_{3,1}]$ can be found by exchanging the indices in the factorial cumulants ($(K)_{m,n}$ becomes $(K)_{n,m}$).

References

[1] Scales N. Resolving fluorescent species by their brightness and diffusion properties using correlated photon counting histograms. Ph.D. Thesis. McGill University; 2013.

[2] Abramowitz M, Stegun IA, editors. Handbook of Mathematical Functions with Formulas, Graphs, and Mathematical Tables. 9th ed. New York: Dover; 1970.

[3] Vergani P, Lockless SW, Nairn AC, Gadsby DC. CFTR channel opening by ATP-driven tight dimerization of its nucleotide-binding domains. Nature. 2005;433:876–880.

[4] Widengren J, Mets U, Rigler R. Fluorescence correlation spectroscopy of triplet-states in solution – a theoretical and experimental study. J Phys Chem. 1995;99:13368–13379.

[5] Palo K, Metz U, Jager S, Kask P, Gall K. Fluorescence intensity multiple distributions analysis: Concurrent determination of diffusion times and molecular brightness. Biophys J. 2000;79:2858–2866.

[6] Melnykov AV, Hall KB. Revival of high-order fluorescence correlation analysis: Generalized theory and biochemical applications. J Phys Chem B. 2009;113:15629–15638.

[7] Hillesheim LN, Müller JD. The dual-color photon counting histogram with non-ideal photodetectors. Biophys J. 2005;89:3491–3507.

[8] Enderlein J, Gregor I. Using fluorescence lifetime for discriminating detector afterpulsing in fluorescence correlation spectroscopy. Rev Sci Instrum. 2005;76:033102.
